# The Complete Genome of *Teredinibacter turnerae* T7901: An Intracellular Endosymbiont of Marine Wood-Boring Bivalves (Shipworms)

**DOI:** 10.1371/journal.pone.0006085

**Published:** 2009-07-01

**Authors:** Joyce C. Yang, Ramana Madupu, A. Scott Durkin, Nathan A. Ekborg, Chandra S. Pedamallu, Jessica B. Hostetler, Diana Radune, Bradley S. Toms, Bernard Henrissat, Pedro M. Coutinho, Sandra Schwarz, Lauren Field, Amaro E. Trindade-Silva, Carlos A. G. Soares, Sherif Elshahawi, Amro Hanora, Eric W. Schmidt, Margo G. Haygood, Janos Posfai, Jack Benner, Catherine Madinger, John Nove, Brian Anton, Kshitiz Chaudhary, Jeremy Foster, Alex Holman, Sanjay Kumar, Philip A. Lessard, Yvette A. Luyten, Barton Slatko, Nicole Wood, Bo Wu, Max Teplitski, Joseph D. Mougous, Naomi Ward, Jonathan A. Eisen, Jonathan H. Badger, Daniel L. Distel

**Affiliations:** 1 Ocean Genome Legacy, Inc., Ipswich, Massachusetts, United States of America; 2 J. Craig Venter Institute, San Diego, California, United States of America; 3 Massachusetts Institute of Technology, Cambridge, Massachusetts, United States of America; 4 New England Biolabs, Ipswich, Massachusetts, United States of America; 5 Architecture et Fonction des Macromolécules Biologiques, UMR6098, CNRS, Universités Aix-Marseille I & II, Case 932, Marseille, France; 6 Department of Microbiology, University of Washington, Seattle, Washington, United States of America; 7 Universidade Federal do Rio de Janeiro, Instituto de Biologia, Ilha do Fundao, CCS, Rio de Janeiro, Rio de Janeiro, Brazil; 8 Department of Environmental and Biomolecular Systems, OGI School of Science & Engineering, Oregon Health & Science University, Beaverton, Oregon, United States of America; 9 Department of Microbiology and Immunology, Faculty of Pharmacy, Suez Canal University, Ismailia, Egypt; 10 College of Pharmacy, University of Utah, Salt Lake City, Utah, United States of America; 11 University of Florida, Gainesville, Florida, United States of America; 12 Department of Molecular Biology, University of Wyoming, Laramie, Wyoming, United States of America; 13 UC Davis Genome Center, University of California Davis, Davis, California, United States of America; University of Hyderabad, India

## Abstract

Here we report the complete genome sequence of *Teredinibacter turnerae* T7901. *T. turnerae* is a marine gamma proteobacterium that occurs as an intracellular endosymbiont in the gills of wood-boring marine bivalves of the family Teredinidae (shipworms). This species is the sole cultivated member of an endosymbiotic consortium thought to provide the host with enzymes, including cellulases and nitrogenase, critical for digestion of wood and supplementation of the host's nitrogen-deficient diet. *T. turnerae* is closely related to the free-living marine polysaccharide degrading bacterium *Saccharophagus degradans* str. 2–40 and to as yet uncultivated endosymbionts with which it coexists in shipworm cells. Like *S. degradans*, the *T. turnerae* genome encodes a large number of enzymes predicted to be involved in complex polysaccharide degradation (>100). However, unlike *S. degradans*, which degrades a broad spectrum (>10 classes) of complex plant, fungal and algal polysaccharides, *T. turnerae* primarily encodes enzymes associated with deconstruction of terrestrial woody plant material. Also unlike *S. degradans* and many other eubacteria, *T. turnerae* dedicates a large proportion of its genome to genes predicted to function in secondary metabolism. Despite its intracellular niche, the *T. turnerae* genome lacks many features associated with obligate intracellular existence (e.g. reduced genome size, reduced %G+C, loss of genes of core metabolism) and displays evidence of adaptations common to free-living bacteria (e.g. defense against bacteriophage infection). These results suggest that *T. turnerae* is likely a facultative intracellular ensosymbiont whose niche presently includes, or recently included, free-living existence. As such, the *T. turnerae* genome provides insights into the range of genomic adaptations associated with intracellular endosymbiosis as well as enzymatic mechanisms relevant to the recycling of plant materials in marine environments and the production of cellulose-derived biofuels.

## Introduction

*Teredinibacter turnerae* is a Gram-negative gamma proteobacterium that has been isolated from the gills of a broad range of wood-boring marine bivalves of the family Teredinidae (shipworms) [1], [2]. This species has been shown to coexist with other as yet uncultivated bacteria as a component of an intracellular endosymbiotic bacterial consortium within specialized cells (bacteriocytes) of the gill epithelium [3], [4]. It displays an unusual combination of properties, being the only aerobic bacterium known to grow with cellulose and dinitrogen, respectively, as its sole carbon and nitrogen sources [2].

The cellulolytic and diazotrophic capabilities of *T. turnerae* suggested two potential roles for this bacterium in the shipworm symbiosis [1]. The first is to produce enzymes that may assist the host in degrading carbohydrate components of woody plant materials (cellulose, hemicellulose, and pectin). Shipworms are the only marine animals known to grow and reproduce normally with wood as their sole source of particulate food [5]. The second is to provide a source of fixed nitrogen to supplement the host's nitrogen deficient diet of wood. The latter function of shipworm symbionts was recently demonstrated experimentally [6].

The capacity to degrade woody plant materials is of interest because these materials are extraordinarily abundant in nature and serve as major reservoirs of carbon and energy [7]. Plant cell walls are typically composed of a complex composite of cellulose, a linear homopolymer of beta 1–4 linked glucose residues, hemicellulose, a decorated heteropolymer of xylose units, and lignins, heterogeneous polymers of aromatic residues. Pectin, a heteropolymer containing alpha 1–4 linked galacturonic acid, is also an important component of plant cell walls. The low solubility of these compounds, and their tendency to form crystalline arrays with complex interconnecting networks of ether and ester linkages, make woody plant materials highly recalcitrant to enzymatic degradation [8].

**Figure 1:**
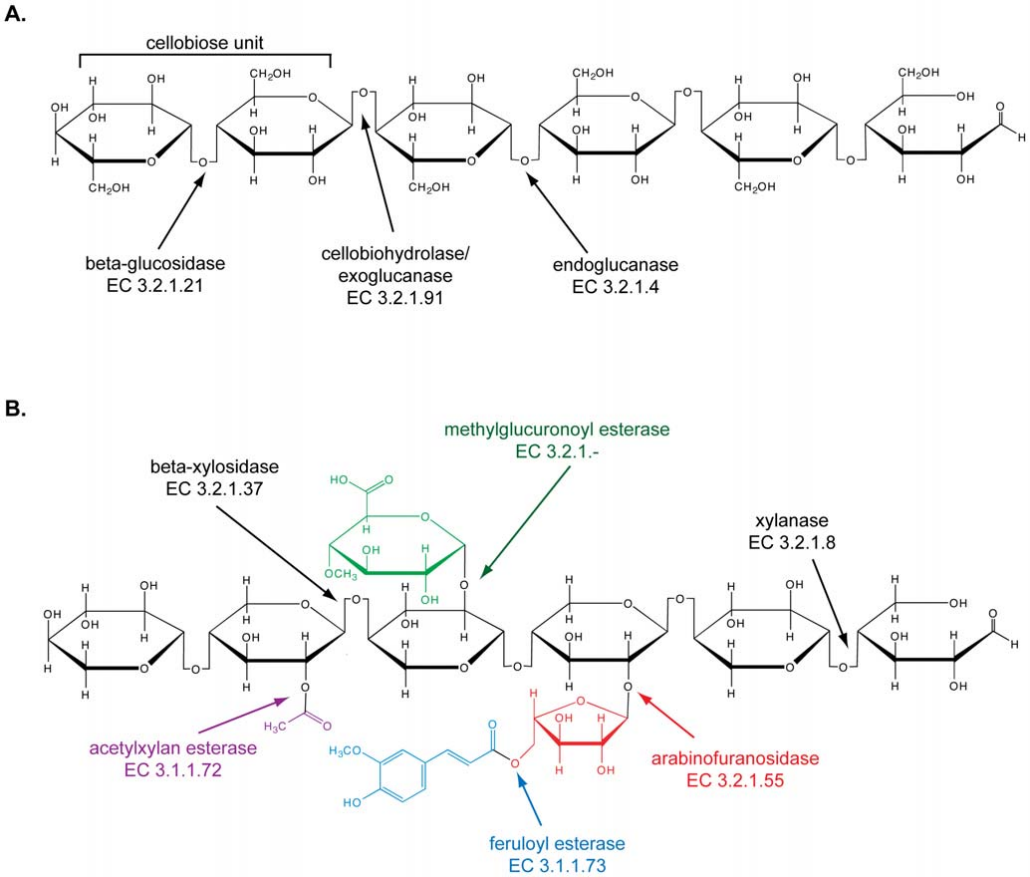
Enzymatic degradation of common components of woody plant materials. Enzymatic components required for the breakdown of cellulose (A) and a hypothetical xylan (B) are shown along with the corresponding EC number designations. Enzymes and the corresponding side-chains cleaved by them are presented in color while substrate backbones and the corresponding enzymes that cleave them are portrayed in black.

The complete degradation of woody plant materials requires numerous enzymes, which in nature are often contributed by multiple microorganisms acting in concert. Cellulose degradation requires at least three types of hydrolytic activities: beta-1,4-glucosidase [E.C. 3.2.1.21], cellobiohydrolase [E.C. 3.2.1.91] and endoglucanase [E.C. 3.2.1.4] (Figure 1A). The depolymerization of hemicellulose requires carbohydrases and esterases that serve to break the xylan backbone and decouple side-chains that may bind to the lignin components of wood (Figure 1B).

The majority of enzymes known to degrade complex polysaccharides belong to a diverse functional category called glycoside hydrolases (GH). GHs are assigned to 112 families (http://afmb.cnrs-mrs.fr/CAZY/) based on nucleotide or amino acid sequence. Many of these families are functionally heterogeneous, containing members that differ in substrate specificities as well as sites and modes of substrate cleavage. In addition to GH activities, degradation of complex polysaccharides may also involve activity of carbohydrate esterases (CE), polysaccharide lyases (PL), and carbohydrate binding modules (CBMs). Moreover, these activities are often found within modular proteins that may contain multiple domains with differing catalytic and substrate binding properties, separated by non-catalytic linker domains. Thus, the composition and organization of polysaccharide degrading proteins may be highly diverse and variable and so exploration of new systems is of considerable interest.

The genome of *T. turnerae* is also of interest as an example of the range of adaptations associated with intracellular endosymbionts of eukaryotes. A characteristic suite of genomic modifications, including reduced genome size, skewed %G+C, elevated mutation rates and loss of genes of core metabolism, has been identified through analysis of genomes of a number of obligate intracellular symbionts [9]. However, this is not the case for *T. turnerae,* which stands as an example of a bacterium that has been observed in nature only as an endosymbiont, but that can be cultivated *in vitro* in a simple defined medium without added vitamins or growth factors. Its comparison to known obligate endosymbionts may therefore be informative.

Here we report the complete genome sequence of the *T. turnerae* strain T7901 (ATCC 39867) isolated from the shipworm *Bankia gouldi*. We examine and discuss the composition and architecture of the genome of this strain with emphasis on systems pertinent to symbiosis and free-living existence.

## Results and Discussions

### Genome features and comparative genomics

#### General genome features

The genome of *Teredinibacter turnerae* T7901 is a single circular molecule of length 5,192,641 bp (50.8% G+C) (Table 1, Supporting Information: Figure S1). The genome encodes 4,690 predicted protein-coding regions. Of these, 3,067 (65.4%) could be assigned a function based on inferred homology, 1026 (21.9%) are hypothetical proteins (no inferred homology to any previously identified proteins), 589 (12.2%) are conserved hypothetical proteins (inferred homology to hypothetical proteins encoded by other genomes), and the remaining 26 (0.5%) appear to be homologues of experimentally confirmed proteins of unknown function. The average ORF length is 973 bp and the average intergenic region is 130 bp. No extrachromosomal elements were detected.

**Table 1:** Comparison of general genome features.

Features	*Teredinibacter turnerae*	*Saccharophagus degradans* 2–40	*Pseudomonas fluorescens Pf-5*	*Cellvibrio japonicus* Ueda107
DNA, total bp	5,192,641	5,057,531	7,074,893	4,576,573
% G+C	51	46	63	52
% Coding	86.5	86.7	88.7	90.5
No. rRNAs	9	6	15	9
No. tRNAs	40	41	71	48
No. ORFs	4690	4008	6144	3790
Mean ORF length	973	1083	1007	1092

#### Phylogenetic affiliations

**Figure 2:**
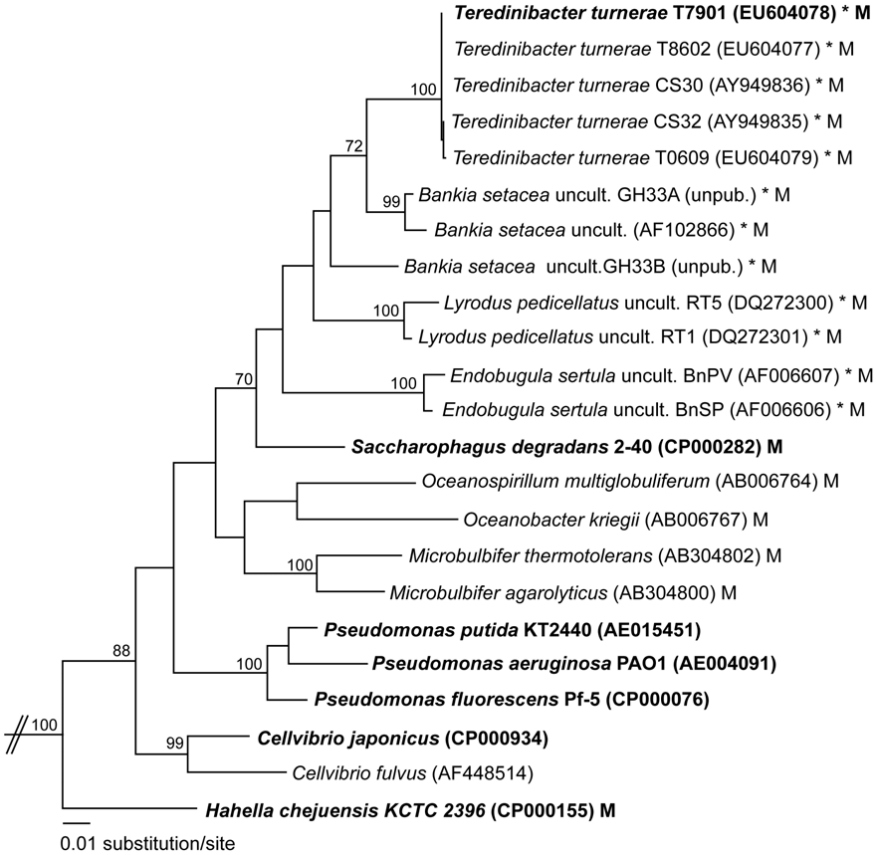
Phylogenetic relationships among *T. turnerae* and selected gamma-proteobacteria. A maximum likelihood (ML) tree based on comparative analysis of 16S rDNA (1,389 aligned characters) inferred using PHYML [99] as implemented in Geneious 3.8.5 (Biomatters Ltd.) is shown for 23 related Pseudomonadaceae. Genbank accession numbers are indicated. Not shown but used in the analysis are 16S sequences from *Escherichia* coli (AE014075 and U00096) and *Salmonella typhimurium* (AE006468 and CP000026). Sequences were aligned manually using MacGDE 2.3 [Linton E: MacGDE: Genetic Data Environment for MacOSX [http://www.msu.edu/~lintone/macgde/] taking into consideration secondary structural information [100]. Bootstrap proportions greater than 70% are expressed to the left of each node as a percentage of 1,000 replicates. The ML tree topology is identical to the consensus tree generated with the same alignment using Mr. Bayes 3.0 (not shown) [101]. Bold taxon labels signify that a complete genome sequence has been determined and is publicly available; asterisks denote symbionts of invertebrates and “M” denotes isolation from marine environments.

Similar strains of *T. turnerae* have been isolated from the gills of 23 species of teredinid bivalves representing 9 host genera collected along the coasts of North and South America, India, Australia and Hawaii [1], [2]. All strains have similar properties and five strains that have been examined by small subunit (16S) ribosomal rRNA sequence analysis are nearly identical with respect to this locus (>99.7% identity: Figure 2).

Phylogenetic analyses of 16S rRNA sequences help to identify closely related bacteria for genomic comparison to *T. turnerae* T7901. These indicate that *T. turnerae* is most closely related to several as yet uncultivated bacterial endosymbionts that have been identified in shipworm gill tissues using cultivation-independent methods (Figure 2) [3], [4]. The closest known free-living (and presumably non-symbiotic) relative is *Saccharophagus degradans* str. 2–40 [10], a marine bacterium isolated from decaying sea grass (*Spartina alterniflora*) in the Chesapeake Bay watershed. This bacterium degrades an unusually broad spectrum of plant, algal, and fungal cell wall components, including >10 classes of complex polysaccharides. Also included within this clade is “*Candidatus Endobugula sertula*” [11], the as yet uncultivated symbiont of the bryozoan, *Bugula neritina*. This symbiont is known to contribute to the chemical defenses of this host species during larval stages by providing a polyketide secondary metabolite (bryostatin) [12], [13], [14], [15] that inhibits predation by fish and that is being considered as a candidate drug for treatment of cancer and dementia. This evokes an additional potential role for *T. turnerae* as a source of secondary metabolites that may contribute to host defenses or maintenance of the symbiotic association.

#### Comparative gene content

**Figure 3:**
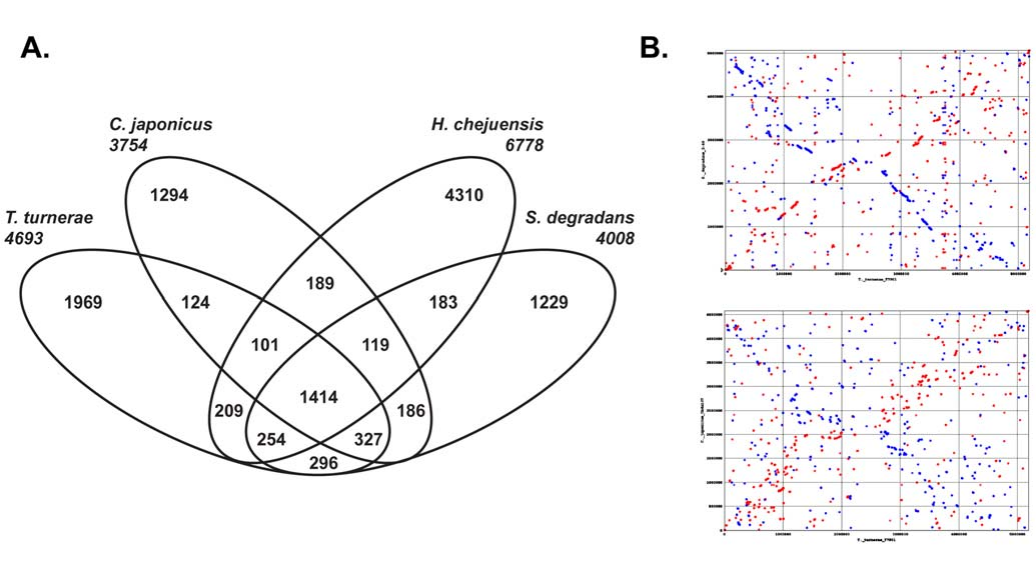
Genomic comparison of *T. turnerae, S. degradans, H. chejuensis* and *C. japonicus*. (A) Venn diagram portraying occurrence of predicted protein coding genes of homologous origin shared among the genomes of *T. turnerae, S. degradans, H. chejuensis* and *C. japonicus*. (B) Synteny of predicted protein coding genes among the genomes of *T. turnerae* (x-axis, top and bottom) and *S. degradans* (y-axis, top) and *C. japonicus* (y-axis, bottom) inferred using PROmer (PROtein MUMmer)[102]. Circular chromosomes are depicted linearly with the origins of replication at map coordinates (0,0). Dots depict location of homologous proteins relative to the origins with red and blue representing homology on the same or opposite strand, respectively.

Recently, complete genome sequences were determined for *S. degradans* str. 2–40 [10] and two other bacterial strains that are closely related to *T. turnerae* T7901 based on 16S rRNA sequences. These are *Cellvibrio japonicus* Ueda107 [16] and *Hahella chejuensis* KCTC 2396 [17]. The shared evolutionary history of these bacterial strains is evidenced by the large number of homologues inferred among predicted open reading frames (ORFs) (Figure 3A) and by the number of these that have best or near best hits among these genomes in total genome BLAST searches. Of the 3,502 predicted proteins in the *T. turnerae* genome that had at least one BLASTP (E<1e-4) hit to a protein encoded by another genome (Combo, DB, Wu et al., unpublished), 1,670 had a best hit to *S. degradans* 2–40, 265 to *C. japonicus* Ueda107, and 85 to *H. chejuensis* KCTC 2396. All other genomes had fewer best hits.

#### Gene organization

**Figure 4:**
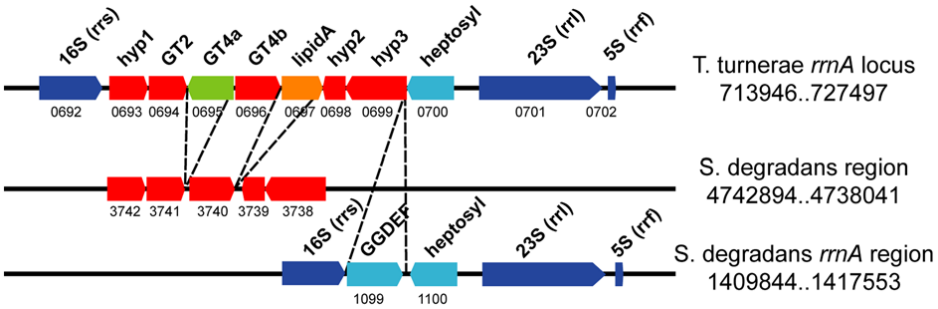
Anomalous organization of ribosomal operon A (*rrnA*) in *T. turnerae* and *S. degradans*. One of three ribosomal RNA operons (*rrnA*) in the *T. turnerae* genome displays unusual organization. In this operon, the small (*rrs*) and large (*rrl*) subunit ribosomal RNA genes are separated by protein coding genes in both *T. turnerae* and *S. degradans* rather than by internal transcribed spacers (ITS) as in most bacteria. All intervening genes have homologues in both genomes. The common location of homologous heptosyltransferase genes in both genomes suggests at least one common ancestral insertion. Ribosomal rRNA genes are depicted in blue. Open reading frames (red, green, orange, and light blue) have been colored to distinguish homologous genes. Dotted lines mark indel boundaries. Chromosomal locus coordinates are indicated to the right of each depicted genome region and unique locus tag numbers are indicated beneath each locus without the preceding GenBank prefixes (TERTU_ for *T.turnerae* or Sde_ for *S. degradans,* respectively).

The genomes of *T. turnerae*, *S. degradans* and *C. japonicus* share similar chromosomal content and organization and display a considerable degree of synteny when protein-coding regions are aligned (Figure 3B). An unusual shared similarity is also observed in the organization of ribosomal genes in *T. turnerae* and *S. degradans*. The ribosomal operons of most bacteria are composed of a 16S rRNA gene (*rrs*) followed by an internal transcribed spacer (ITS1), a 23S rRNA gene (*rrl*), a second internal transcribed spacer (ITS2) and finally a 5S rRNA gene (*rrf*). In addition to ribosomal operons with this canonical organization, the genomes of *T. turnerae* and *S. degradans* each contain a single occurrence of *rrs* and *rrl* genes that are separated by putative protein coding sequences (Figure 4) rather than a typical ITS1. The ITS1 region of most known gamma-proteobacteria ranges from ∼250–1,000 nucleotides in length and encodes one or two tRNA genes but does not contain protein-coding genes [18]. In contrast, *S. degradans* contains two putative protein-coding sequences within this region while the comparable region in *T. turnerae* encodes eight.

In both genomes, the intervening ORFs most proximal to the *rrl* are homologous putative heptosyltransferase genes. Moreover, the remaining seven ORFs embedded in the ITS1 of *T. turnerae* also have homologues elsewhere in the *S. degradans* genome, five of which are syntenic in both genomes. This gene organization strongly suggests that a common ancestral insertion resulted in the proximity of *rrl* and heptosyltransferase genes in both genomes and that this event was likely followed by at least four rearrangement events (Figure 4) to arrive at the extant gene orders. The large size of these insertions and alternating orientation of the contained ORFs suggest that these *rrs* and *rrl* genes are no longer contained within a single transcriptional unit in either genome.

### Polysaccharide degradation systems of *Teredinibacter turnerae*

#### Wood specialization

Like that of its closest known relative, *S. degradans*, the *T. turnerae* genome is notable for containing an unusually large number of protein domains involved in the degradation of complex polysaccharides, including glycoside hydrolases (GH), carbohydrate esterases (CE), pectin lyases (PL), and carbohydrate binding modules (CBM). However, in contrast to *S. degradans,* which is a generalist capable of degrading more than 10 types of plant, algal, animal and fungal polysaccharides [19], [20], [21], the *T. turnerae* genome lacks enzyme systems for degradation of common marine polysaccharides including agar, alginate, and fucoidan and has only comparatively sparse representation of chitinase (two vs. seven in *S. degradans*) and laminarinase (six vs. ten in *S. degradans*) genes. Enzymes for degradation of the fungal polysaccharide pullulan are also absent in *T. turnerae*. Instead, the gene content of the *T. turnerae* genome suggests a high degree of specialization for degrading polysaccharides associated with woody plant materials, including cellulose, xylan, mannan, galactorhamnan and pectin.

The *T. turnerae* genome encodes a total of 123 ORFs dedicated to processing complex polysaccharides. Of these ORFs, 95 encode GH domains, 4 encode PL domains, 18 encode CE domains, and 4 encode both GH and CE domains (Supporting Information: Tables S1, S2, and S3). The total number of GH domains in *T. turnerae* is similar to that of *S. degradans* [10] and *C. japonicus* [16], which places *T. turnerae* among the top 5% of over 750 bacteria with sequenced genomes (BH & PMC, unpublished). Notably, *T. turnerae* possesses a high number of CE domains per single genome among these organisms (see Table 2), suggesting a considerable investment in capacity for degrading hemicelluloses.

**Table 2:** Summary of carbohydrate binding and catalytic domains found per genome or metagenome.

	*Teredinibacter. turnerae*	*Saccharophagus degradans*	*Ccllvibrio japonicus*	*Nasutitermes Community*
Glycoside hydrolases (GHs)	101	130	122	704
Polysaccharide lyases (PLs)	5	33	14	10
Carbohydrate esterases (CEs)	22	15	19	n/d
Carbohydrate binding modules (CBMs)	117	136	93	10

**Figure 5:**
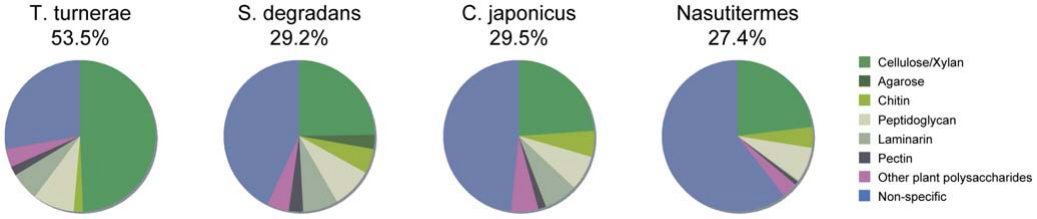
Prevalence of GH domains as a function of substrate specificity. The genome of *T. turnerae* contains a larger proportion of glycoside hydrolase (GH) domains with specificity for major wood components (cellulose, xylan, mannans, and rhamnogalactans) than do other compared genomes and metagenomes. GH domains are sorted by known substrate specificity and presented as a fraction of the total number of GH domains per genome. Substrate specificities are coded as follows: green = cellulose/xylan, (GH families 5, 6, 8, 9, 10, 11, 12, 44, 45, 51, 52, 62, and 74), dark green = agarose (GH families 50 and 86), light green = chitin (GH families 18, 19, and 20), light grey = peptidoglycan (GH families 28 and 105), dark grey = laminarin (GH families 16 and 81), black = pectin (GH families 28 and 105), purple = other woody plant cell wall polysaccharides (GH families 26, 53, and 67) and blue = GH domains with other specificities or specificities not uniquely predicted by family designation. Proportions are expressed as a fraction of the total number of GH domains found in the genomes of *Teredinibacter turnerae* (101), *Saccharophagus degradans* (130), *Cellvibrio japonicus* (122), and *Nasutitermes* termite hindgut metagenome community (704) respectively. The fraction of total GH domains with specificity toward cellulose and xylan is indicated below each species name.

The diversity of GH domain families represented in the *T. turnerae* genome is also comparable to those of the other cellulolytic bacteria. The *T. turnerae* genome encodes catalytic domains representing 38 different GH families. This compares to 38 in *S. degradans*, 42 in *C. japonicus*, and 44 in a recently characterized metagenome derived from a community containing over a thousand bacterial types in the hindgut of the termite *Nasutitermes sp* [22]. (Figure 5).

Although the absolute number and diversity of GH domains is similar, the proportion of GH domains predicted to have activity against components of woody plant materials (cellulose, xylan, mannan, and rhamnogalactans) in the *T. turnerae* genome (Figure 5) is 53%, nearly twice that of *S. degradans* (29%), *C. japonicus* (29%) or the *Nasutitermes sp*. hindgut community (27%). Indeed, seven GH families (GH6, GH9, GH10, GH11, GH44, GH45, and GH62) are represented by at least twofold more domains in *T. turnerae* than in *C. japonicus* or *S. degradans*. Each of these has predicted activity against cellulose or xylan.

#### Multidomain and multicatalytic enzymes

**Figure 6:**
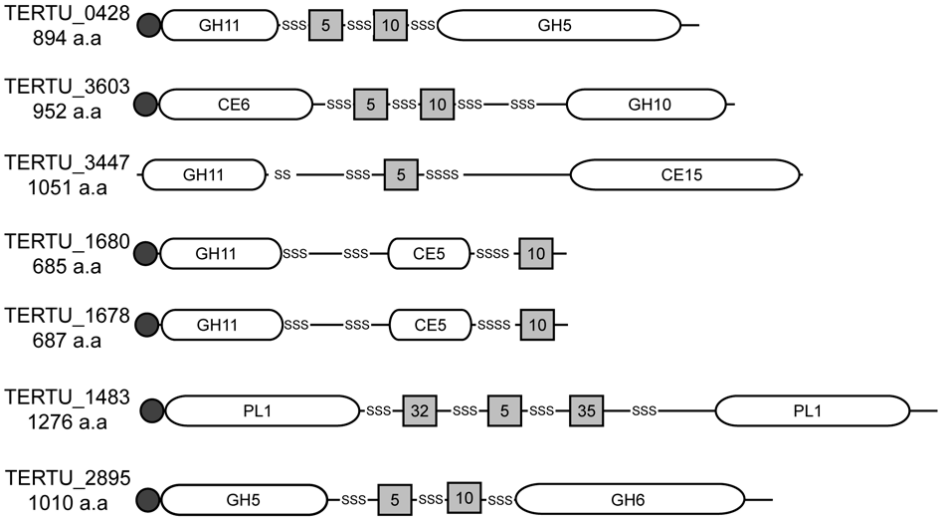
Multicatalytic carbohydrate-active enzymes. The genome of *T. turnerae* encodes seven multicatalytic enzymes (enzymes containing multiple catalytic domains with distinct predicted substrate specificities). Domain architecture is depicted in schematic form. Key: glycoside hydrolases (GH), carbohydrate esterases (CE), polysaccharide lyases (PL), catalytic domains (white rounded rectangles), carbohydrate binding modules (grey squares), secretion signals (dark circles), and polyserine linker regions (“SS”). Numbers specify domain families (http://afmb.cnrs-mrs.fr/CAZY/).

The *T. turnerae* genome is also unusual in the number of multicatalytic enzymes (single proteins with multiple catalytic domains, each with distinct predicted catalytic activities) that it encodes and in the domain composition of these enzymes. While multidomain carbohydrases are common, most are composed of a single catalytic domain plus one or more carbohydrate-binding modules or other small domains of unknown function. It is relatively unusual for such enzymes to include multiple catalytic domains and still less common for those domains to differ in substrate specificity (BH & PMC, unpublished). A small number of such multifunctional, multidomain glycosidases have been characterized experimentally, including a cellulase (CelAB) from *T. turnerae* [23], a cellulase (Cel5A) and a chitinase (ChiB) from *S. degradans* [21], [24], and an endoglycosidase from *Enterococcus faecalis* [25]. The *T. turnerae* genome encodes seven such multicatalytic enzymes (Figure 6).

The advantages of co-localizing distinct catalytic domains within a single protein are unknown, but may reflect the special problems encountered by organisms that depend on extracellular degradation of complex insoluble substrates like wood. For example, cellulose, a major constituent of wood, requires both endoglucanase and cellobiohydrolase activities to convert the insoluble polymer efficiently into soluble sugars that can be transported across the cell envelope. Combining endoglucanase and cellobiohydrolase activity along with carbohydrate binding modules in a single molecule may ensure proximity of these complementary catalytic domains, and therefore enhanced activity, while also preventing diffusion of these proteins away from their insoluble substrates.

The complete hydrolysis of hemicellulose also requires a combination of hydrolytic activities, including glycoside hydrolases and esterases. Hemicellulose in softwoods and hardwoods are predominantly composed of *O*-acetyl-(4-*O*-methylglucurono)xylan [26]. In contrast, arabinoxylan is the major heteroxylan in grasses. Consistent with its proposed specificity for degrading woody plant materials, *T. turnerae* encodes four multicatalytic hemicellulases, each of which is predicted to be bifunctional. Three encode both xylanase and acetylxylan esterase domains (TERTU_3603, TERTU_1680, and TERTU_1678), and a fourth encodes xylanase and methylglucuronoyl esterase domains (TERTU_3447). Although a few multicatalytic enzymes with the former combination of specificities have been described previously, e.g. [27], [28], this is the first report of the latter combination.

Another proposed advantage of such multicatalytic proteins is that they may promote intramolecular synergism [23]. For example, endoglucanases and cellobiohydrolases are thought to display synergism because the former produces substrate (reducing ends) that can be degraded by the latter. Indeed, such synergism has been observed between naturally co-occurring endoglucanases and exoglucanases or accessory enzymes such as cellodextrinases and cellobiases in *Fibrobacter* [29], *Clostridium* [30], *Cellulomonas* [31] and fungi [32]. Moreover, cellulases and cellulase complexes (synthetic cellulosomes) engineered to contain multiple enzymatic specificities also display synergistic activity against cellulose [33] and complex plant substrates [34], [35].

The co-localization of multiple activities within a single protein may also have advantages specific to symbiosis. It is thought that enzymes produced by shipworm symbionts are transported by an as yet unknown mechanism from the shipworm's gills, where the symbionts are found, to the digestive system where lignocellulose degradation is thought to occur [36]. Therefore, combining multiple specificities within a single protein may simplify the task of protein transport.

#### Carbohydrate binding modules

In addition to a diversity of carbohydrate-active catalytic domains, the *T. turnerae* genome also contains 117 domains predicted to bind carbohydrates, second only to *S. degradans*. Most abundant are those predicted to bind crystalline cellulose (CBM2 and CBM10), which account for nearly 50% of all CBMs in the genome. These are also the most abundant CBM types in *S. degradans* and *C. japonicus*. However, relative to these other genomes, *T. turnerae* is enriched in the xylan binding CBM family 22 (seven in *T. turnerae*, one in *S. degradan,s* and none in *C. japonicus*), consistent with a specialization for degradation of hemicellulose or heteroxylan components of wood.

Interestingly, seventeen ORFs in the *T. turnerae* genome contain CBMs that either lack associated catalytic domains or lack domains with putative carbohydrase activity (GH, CE, or PL enzyme functions, Supporting Information: Table S4). Indeed two of these link CBMs to domains predicted to function as serine proteases. The functions of these unassociated CBMs and unusual hybrids are unknown, but may involve modification of the surface of insoluble woody substrates, or modification of other proteins/enzymes bound to these surfaces.

#### Polyserine linker domains

The linker regions that join carbohydrase domains and CBMs in *T. turnerae* are also unusual, being uncharacteristically long and comprised nearly entirely of serine residues. Linkers found in other carbohydrases commonly consist of repeating proline, threonine and glycine residues. While the functional significance of polyserine linkers (PSLs) is unknown, they appear to be characteristic of the carbohydrases of *T. turnerae* and its closest relatives *C. japonicus* and *S. degradans* [37], [38].

The *S. degradans* genome encodes 46 carbohydrate active proteins that contain PSLs. On average, these are composed of 80% serine residues and are 39 residues in length [38]. Similarly, *T. turnerae* encodes 42 carbohydrate active proteins containing PSLs with an average serine content of 83% and average length of 44 residues. Approximately one-third of GH and CE domain-containing proteins are linked by PSLs to one or more CBMs, as are most (75%) PL domains. Of the multidomain glycoside hydrolases, >80% of these also exhibit polyserine linker regions while all of the multidomain carbohydrate esterases and polysaccharide lyases contain PSLs.

### Bioinformatics evidence for secretion system gene clusters

In Gram-negative bacteria, several secretion systems are available to catalyze the extracellular translocation of proteins and genetic material. For some secretion systems, (e.g. the type II secretion system, or T2SS), proteins must first be exported across the inner membrane to the periplasm using the so-called “conventional” or “broad-specificity” Sec pathway before translocation across the outer membrane. Other secretion systems use complex multi-component protein assemblies that bypass the Sec pathway requirement and directly translocate proteins from the cytoplasm to the extracellular milieu. Specialized secretion systems, such as types III, IV, and VI (T3SS, T4SS, and T6SS) are often hallmarks of intracellular bacterial pathogens and symbionts [39], and many of the secretion system substrates, termed “effectors,” have been shown to modify or disrupt host cell function [40], [41], [42].

#### Type 2 secretion systems

Genome sequence suggests that both Sec and Sec-independent (twin-arginine, Tat) pathways contribute to protein translocation across the inner member in *T. turnerae* (for reviews, see [43], [44], [45]). All essential components of the Sec translocase encoded by *secA, secY, secE*, and *secG*, as well as gene products of *secD, secF, yajC* and *yidC* that are peripherally associated with the translocase have been identified. Both the co-translational/SRP pathway (involving the Ffh/SRP54 riboprotein and the FtsY receptor) and the postranslational pathway (involving the SecB chaperone) of translocase targeting appear to be present. Over 20% of the predicted proteins in the *T. turnerae* genome were identified with N-terminal signal peptides by SignalP 3.0 [46] and are predicted to be Sec pathway substrates. Proteins deposited in the periplasm by the Sec and Tat pathways may be translocated through the outer membrane by the T2SS, also known as the general secretory pathway. All known essential components of T2S are arranged in a single large operon (*gspCDEFGHIJKLMN*) in the *T. turnerae* genome, as was previously observed in *S. degradans*.

#### Type 3 and type 4 secretion systems

Secretion pathways are an important means by which intracellular symbiotic and pathogenic bacteria modulate interactions with their hosts. Two of these, the T3 and T4 secretion systems, are known to play important roles in many plant and animal symbionts and pathogens [47]. For example, T3 and T4 pathways are involved in the establishment of symbiosis by the tsetse fly endosymbiont *Sodalis glossinidius* and the plant endosymbiont *Mesorhizobium loti* [48], [49], respectively. Surprisingly, elements encoding T3 or T4SSs are absent from the genome of *T. turnerae*.

#### Type 6 secretion systems

Interestingly, however, elements of the T6SS, a Sec-independent secretion system only recently described in Gram-negative bacteria [50], [51], were identified in the genome of *T. turnerae* (TERTU_1668-TERTU_1639). Reports indicate that T6S can perform a myriad of functions including promoting biofilm formation, and attenuating or enhancing virulence [50], [52], [53], [54], [55]. A protein secreted from *Vibrio cholerae* in a T6SS-dependent manner can crosslink actin in macrophages [56], thereby indicating that T6SSs are likely to directly translocate protein(s) into host cells. The fact that *T. turnerae* resides inside host cells as an endosymbiont and lacks T3 and T4 secretion suggests that T6S might be a central mechanism for the initiation and maintenance of the symbiotic interaction. Indeed, it has been demonstrated that T6S is a determining factor for host-specificity in the symbiont *Rhizobium leguminosarum* [57], [58].

**Figure 7:**
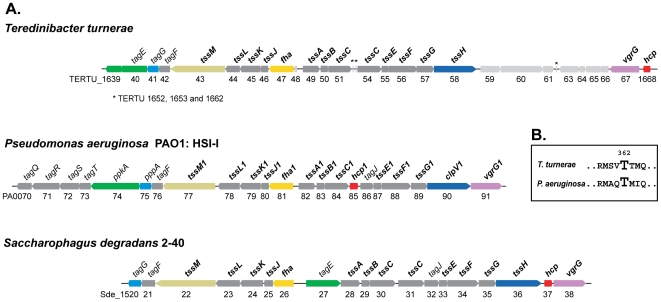
Type 6 secretion system (T6SS) gene clusters. (A) Schematic representation of T6SS clusters of *T. turnerae*, *P. aeruginosa* and *S. degradans*. The T6SS units are arranged so that the *fha* ortholog of each species is central. Genes are identified by locus tag numbers (below) and according to the standardized nomenclature for T6SS proposed by Shalom *et al.* [103] (above): *tss*; core components in all T6SS units; *tag*: tss-associated genes which are present in T6SS clusters in more than one bacterial species. Genes highlighted in color are discussed in the text and homologous genes are represented by the same color. Genes indicated with light grey have not been clearly linked to T6S function or are not widely conserved in T6S loci. T6SS core genes [104] are indicated in bold type. (B) Partial sequence alignment of the FHA domain-containing proteins involved in posttranslational regulation of T6S in *T. turnerae* and *P. aeruginosa*. The critical phosphorylation site (Thr362) is conserved in the *T. turnerae* protein.

In order to gain greater insight into the potential function of T6S in *T. turnerae*, we compared its T6SS locus structure and gene content to that of *P. aeruginosa* HSI-I, a well-characterized T6S locus, and to the T6S locus of its close relative, *S. degradans* (Figure 7). The T6S gene cluster in *T. turnerae* appears to encode all known essential proteins of the secretory apparatus (E value: <1e-10) (Figure 7A). Among these are genes encoding proteins with homology to IcmF (TssM) and an AAA^+^-family ATPase (TssH), which are hallmarks of the T6SS [59]. The T6SS generally translocates at least two proteins: haemolysin-coregulated protein (Hcp) and the valine-glycine repeat protein G (VgrG) [60], [61], [62], [63]. Genes encoding these apparent substrates of the system are present in the T6SS cluster of *T. turnerae*, and furthermore, two other putative *vgrG* genes (TERTU_3731 and TERTU_2226) are located outside of the unit.

Overall, these loci are highly similar, with 17 and 19 of the 22 *P. aeruginosa* HSI-I genes conserved in *T. turnerae* and *S. degradans*, respectively. Surprisingly, our analyses indicated that the overlap in T6S gene between these organisms is not restricted to those genes that are widely conserved in other T6SSs. Moreover, detailed comparisons of the proteins putatively involved in T6S activation in *T. turnerae* with those of *P. aeruginosa*, provided evidence that not only are essential proteins of this pathway conserved and likely to be functional, but also, that the mechanism of initiation and signal propagation may be as well. Sequence alignment of Fha1 with TERTU_1647 (*fha*) showed that the site of Fha1 phosphorylation (Thr362) is conserved in the *T. turnerae* protein (Figure 7B). Likewise, similar to PpkA, a C-terminal periplasmic extension was observed in its *T. turnerae* ortholog (TERTU_1640). As is typical in T6S kinases, there is no significant sequence similarity of this region between the species (data not shown).

#### Secretion and localization of carbohydrate-active proteins

As observed previously in *C. japonicus* and *S. degradans*, the majority of carbohydrate active enzymes encoded by *T. turnerae* appear to be substrates of the type II secretion system (T2SS). Of the 99 GH and the 22 CE genes, 73 and 14 proteins respectively are predicted to have an N-terminal signal peptide for secretion. Notably, four out of five candidate beta-glucosidases do not have T2SS consensus signal peptides, suggesting that cellobiose and/or larger polymers may be targeted by a phosphotransferase transport system for further degradative processing in the cytoplasm. Consistent with this notion, a candidate cellobiose phosphotransferase gene (TERTU_3237), and three candidate cellobiose phosphorylase genes (TERTU_2767, TERTU_2762, and TERTU_0851) have been identified in *T. turnerae*.

Over 180 proteins encoded by the *Teredinibacter* genome are predicted to be lipoproteins (LipoP 1.0 program [64]) and so are likely to become anchored to the outer face of the outer membrane. Of these, 23 are associated with polysaccharide degradation. Localization of lignocellulose active proteins to the outer membrane may provide some of the functional advantages to Gram-negative bacteria that cellulosomes provide for Gram-positive bacteria.

#### Experimental analysis of the T. turnerae secretome

**Figure 8:**
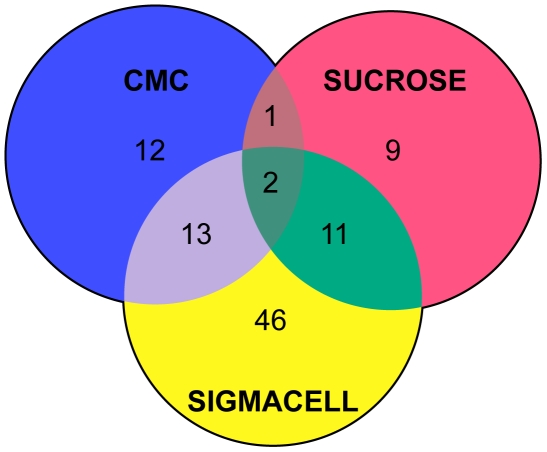
Secretome of *T. turnerae*. Venn diagram depicting proteins expressed during growth with indicated carbon sources. Numbers in non-overlapping regions enumerate proteins that were uniquely expressed and secreted under the indicated condition. Numbers in overlapping regions enumerate proteins expressed and secreted under multiple growth conditions.

A total of 123 proteins were identified under the assayed growth conditions (Figure 8). Based on sequence data, most were predicted to be secreted proteins or lipoproteins, although a small proportion had no predicted N-terminal signal peptides and were presumed to be cytoplasmic proteins released by cell lysis. The number of proteins detected in spent medium after growth on SigmaCell (72 proteins), was more than three times greater than that observed after growth on carboxymethyl cellulose (25 proteins) or sucrose (23 proteins), possibly reflecting the greater demands of degrading insoluble polysaccharides. Two proteins, a predicted cellodextrinase (TERTU_0427) and a xylose isomerase (TERTU_4666), were expressed under all conditions tested. Several carbohydrases, including three endoglucanases (TERTU_2893, TERTU_3565, and TERTU_4054), were detected only with SigmaCell as the sole carbon source (Table 3). Detection of cellodextrinase and xylose isomerase under all growth conditions suggests that, as in other woody biomass-degrading microorganisms, expression of certain enzymatic activities required for cellulose and xylan metabolism may be linked. This may be resolved by further transcriptomic and/or proteomic analyses of *T. turnerae* grown on purified cellulose and hemicellulose components. It should be noted that the methods used here target secreted proteins and may not efficiently detect cytoplasmic or periplasmic proteins, or proteins that bind irreversibly to insoluble substrates.

**Table 3:** Examples of *T. turnerae* secreted carbohydrate active enzymes and associated proteins*.

ORF	Predicted Function	Domain Architecture	AA	MW (kD)	LOC	SUC	CMC	SIG
TERTU_2703	carbohydrate esterase	CE3-CBM10-CBM2	486	49	S	−	−	+
TERTU_0645	endoglucanase	GH9-CBM10-CBM2	876	91	S	+	−	+
TERTU_0607	endoglucanase	GH9	580	63	S, L	−	+	−
TERTU_0427	cellodextrinase	CBM5-CBM10-GH5	699	75	S	+	+	+
TERTU_0046	chitin/cellulose binding protein	CBM33-CBM10	332	35	S	−	+	+
TERTU_4506	xylanase	GH8	436	49	S, L	−	+	+
TERTU_4054	endoglucanase	GH44	556	63	S	−	−	+
TERTU_3603	acetylxylan esterase-xylanase	CE6-CBM5-CBM10-GH10	952	100	S	−	−	+
TERTU_3565	endoglucanse	CBM2-GH5	628	68	S	−	−	+
TERTU_2893	endoglucanse	GH9-CBM3-CBM5-CBM10	875	93	S	−	−	+
TERTU_2567	carbohydrate binding protein	CBM10-CBM5	1324	432	S	−	−	+
TERTU_2546	xylanase	CBM2-CBM10-GH10	629	66	S	−	−	+
TERTU_1675	beta-1,3-glucanase	GH16-CBM6	338	37	S	−	−	+
TERTU_1599	xylanase	GH10-CBM6-CBM22-CBM22	955	103	S	+	−	+
TERTU_1498	alpha-glycosidase	GH31	977	110	S, L	−	−	+
TERTU_0768	alpha-L-arabinofuranosidase	GH51	515	58	S	−	−	+
TERTU_0766	carbohydrate binding protein	CBM13-NPP1	392	43	S	+	−	+
TERTU_2895	endo- and exoglucanase	GH5-CBM5-CBM10-GH6	1010	106	S	+	+	−

* As determined by 2-D LC MS/MS on spent culture medium (see methods). Abbreviations: number of amino acid residues (AA), molecular weight (MW), predicted protein localization (LOC), secreted (S), lipoprotein (L), sucrose (SUC), carboxymethycellulose (CMC), and SigmaCell (SIG).

### Nitrogen metabolism

#### Nitrogen fixation

Wood is a carbon-rich but nitrogen-poor substrate. Therefore, organisms that utilize wood as food may benefit from alternate sources of nitrogen nutrition such as nitrogen fixation. The genome of *T. turnerae* revealed a complete set of nitrogen fixation genes (*nif*) organized in three main clusters that span nearly 60,000 bp, or 1% of the genome. The first cluster contains nitrogenase accessory and regulatory genes including *nifQ, nifBAL*, and the electron transport complex genes *rnfABCDGE*. The second cluster contains the structural nitrogenase genes encoded by the *nifHDKT* operon. The third cluster contains genes *nifENX* whose gene products function to synthesize nitrogenase molydenum-iron cofactors, as well as *nifUSVPWZM* whose gene products also function in nitrogen fixation. The organization of the later gene cluster is particularly similar to the *nif* gene arrangement in strains of *Azotobacter*, diazotrophic Gram-negative gamma-proteobacteria related to Pseudomonads. Consistent with this observation, sequence analysis of the *T. turnerae* NifH protein revealed that the closest related sequence is the *nifH* gene from *Pseudomonas stutzeri*. These data, along with the absence of *nif* genes in *S. degradans, H. chejuensis*, and *C. japonicus*, are consistent with the notion that the *nif* cluster in *T. turnerae* was acquired via horizontal gene transfer from a *Pseudomonas*-like bacterium, as has been proposed for *nif* genes in other microbes [65].

#### Nitrogen assimilation

In addition to genes involved in nitrogen fixation, about 40 genes in the genome of *T. turnerae* are predicted to function in nitrogen assimilation. The majority of these are dedicated to urea metabolism and transport. Specifically, an operon containing urease and accessory genes (*ureABCEFG*) is flanked by two operons (*urtABCDE*) dedicated to the energy-dependent transport of urea. Urease and urea transporter genes are also found in the genome of *S. degradans* and *Hahella chejuensis*, suggesting that urea metabolism may be common to this lineage of marine bacteria.

The *T. turnerae* genome also encodes eight genes (TERTU_3348, TERTU_0619, TERTU_4234, TERTU_3878, TERTU_3828, TERTU_2171, TERTU_1871, and TERTU_1053) predicted to encode carbon-nitrogen (C-N) hydrolases. This prediction is supported by the presence in each of these genes of a diagnostic triad of conserved amino acid residues (Glu-Lys-Cys) required for attack on cyano- or carbonyl carbon substrates. C-N hydrolases are members of a protein superfamily containing 13 families, one of which is responsible for nitrilase activity [66]. A putative nitrilase function was assigned to a *T. turnerae* C-N hydrolase gene, TERTU_4234 based on protein identity (81%) to a functionally characterized nitrilase (AAR97393) [67]. TERTU_4234 is part of a seven-gene operon named Nit1C (Supporting Information: Figure S2) that is evolutionarily conserved from cyanobacteria through myxobacteria. It has been proposed that Nit1C genes may be involved in detoxification of xenobiotic compounds from plants and microbes [67] and in the production of a PKS/NRPS hybrid antibiotic cystothiazole A (Feng et al., 2005). Nitrilases are commercially important in the production of acrylamide and other fine chemicals [68], [69].

### Quorum sensing

Many symbiotic proteobacteria rely on quorum sensing, a mechanism of bacterial population density-dependent gene regulation, to structure their host-associated microbial communities. The genome of *Teredinibacter* does not appear to encode homologues of known LuxI or LuxM-type AHL synthases, which are commonly required for the synthesis of N-acyl homoserine lactone quorum sensing signals. However, it is worth noting that *Nitrosomonas europea* produces three AHLs, even though the genome of *N. europea* does not contain a canonical *luxI* or *luxM* [70]. A LuxR-type protein (TERTU_2802), weakly homologous to known AHL receptors, is encoded within the *Teredinibacter* genome. It is not yet known whether this “orphan” LuxR protein may function as a receptor for AHLs produced by other bacteria. Some gamma-proteobacteria (*Salmonella, E. coli, Klebsiella*) do not produce AHLs themselves, but have functional AHL receptors that detect AHLs produced by other bacteria within a microbial community [71]. The AHL receptor gene (*sdiA*) in these bacteria is considered to be a horizontal acquisition that followed a loss of the conserved *luxI-luxR* cluster [71]. The genome also does not appear to encode a homologue of LuxS, a synthase of a furanone AI-2 signal [72].

In addition to AHL- or AI-2- mediated quorum sensing, social behaviors in all gamma-proteobacteria are mediated by the orthologues of the GacS/GacA two-component system [73], [74]. The genome of *T. turnerae* encodes a GacS orthologue (TERTU_1191) and a GacA orthologue (TERTU_2408). The predicted periplasmic loop of GacS and its cytoplasmic linker domain (responsible for the interactions with the yet unknown signal) [75] appear to be the least conserved in this family of orthologues. It is, therefore, not yet clear whether GacS*_T.t._* responds to the same self-produced signal that was initially characterized in pseudomonas [75]. Similar to other orthologues, GacS*_T.t._* contains H302, D720 and H878, predicted to function in autophosphorylation and phosphotransfer to GacA. The GacA is most likely functional since it contains D54 (a predicted phosphorylation site) and the highly conserved amino acid residues (D8-D9, P58-I61, T82-D86) that are predicted to interact with D54. The GacS/GacA-mediated signal transduction in gamma-proteobacteria requires an RNA binding protein CsrA ( = RsmA), which interacts with the small regulatory RNAs controlled by GacS/GacA. The genome of *T. turnerae* is unusual in that it appears to contain two CsrA homologues (TERTU_2809 and TERTU_2436). TERTU_2809 was most similar to the annotated *csrA (rsmA)* from *Saccharophagus degradans, Cellvibrio japonicus,* and *Pseudomonas mendocina.* TERTU_2436 does not appear to have orthologues in these related bacteria and is most similar to TERTU_2809. The GacS/GacA-Csr system also contributes to the regulation of genes involved in utilization of various carbon sources and secondary metabolism [75].

### Secondary metabolism

**Figure 9:**
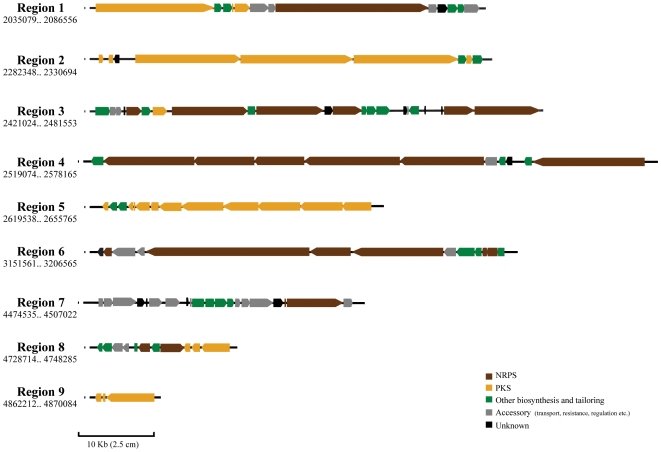
Secondary metabolism gene clusters of *T. turnerae*. Predicted secondary metabolite gene clusters in the genome of *T. turnerae* T7901. Regions are shown in order of distance from the origin of replication. Sequence coordinates of each region are indicated beneath the region number. NRPS: nonribosomal peptide synthetase, PKS, polyketide synthase.

The *T. turnerae* genome contains nine gene clusters predicted to encode secondary metabolite pathways, including multifunctional and modular polyketide synthase (PKS) and non-ribosomal peptide synthase (NRPS) enzymes, which are typically involved in the production of bioactive molecules (Figure 9). Clusters were delimited as groups of genes with homologues among secondary metabolite pathways. The clusters range in size from 8 Kb (Region 9) to 74 Kb (Regions 3 and 4) and contain many large ORFs, such as TERTU_2858, a remarkable ∼22 Kb in length (Figure 9). Modular PKS and NRPS are enzymes that devote separate modules, consisting of groups of catalytic domains, to each elongation step of the growing molecular chain. Thus, size of these enzymes is correlated to the size of the metabolites. The size and complexity of some of the clusters suggests that large, complex metabolites are likely to be produced by *T. turnerae*. The combined putative secondary metabolite pathways account for approximately 380 Kb, or nearly 7% of the *T. turnerae* genome. Thus, the fraction of the genome devoted to secondary metabolism in *T. turnerae* is comparable to that found in several *Streptomyces* species considered to be secondary metabolism specialists [76], [77].

A detailed analysis of the secondary metabolome of *T. turnerae* T7901 is beyond the scope of this work and will be presented in a separate manuscript; an overview is presented here. Three of the clusters, 4, 6 and 7, are NRPS clusters, without PKS elements. Region 7 contains genes homologous to biosynthesis of a enterobactin-like catecholate siderophore, including a Dhb/Ent-F-like NRPS (TERTU_4067) and a ent*CEBA*-like operon required for production (through conversion of the aromatic amino acid chorismate) and activation of DHBA (TERTU_4059–4062) (Figure 9, Region 7 ORFs in green) [78]. This region, predicted to be responsible for siderophore biosynthesis and iron transport, is the only one among the 9 detected clusters for which prediction of the compound class was possible based on BLAST analysis of ORFs. Completing the *T. turnerae* secondary metabolome are one small (Region 9) and two large (Region 2 and 6) modular PKS gene clusters. Regions 1, 3 and 8 are mixed clusters, containing genes homologous to both NRPS and modular PKS functions (Figure 9).

The large number and high complexity of NRPS and PKS clusters observed in *T. turnerae* is in sharp contrast to that seen in the sequenced genome of *S. degradans*. In an analysis of *S. degradans* 2–40 genomic database (http://genome.jgi-psf.org/finished_microbes/micde/micde.home.html), we detected 5 *loci* containing ORFs coding for hypothetical PKS/NRPS enzymes. These regions, which total ∼73 Kb in size, represent 1.43% of the *S. degradans* 2–40 genome. Only 3 of the putative enzymes (Sde_0688, Sde_0689 & Sde_3725) exceed 2000aa in length and only one (Sde_3725) is structurally more complex than a mono-modular type I PKS. The predicted siderophore biosynthesis cluster in *S. degradans* is smaller (17,690 bp) than its *T. turnerae* (32,073 bp) counterpart. It remains to be determined whether the greater complexity of these genes in *T. turnerae* is correlated with host association.

Comparative analyses suggest that the NRPS and PKS clusters of *T. turnerae* may have phylogentic origins distinct those of *S. degradans*. As previously mentioned nearly half of all ORFs in the *T. turnerae* genome with significant BLAST hits to another sequenced genome have best hits to homologues in *S. degradans*. This is not the case for PKS/NRPS clusters. For example, regions 1 and 2 are PKS and/or NRPS enzymes with significant similarities (45–50%) to recently characterized enzymes of *Bacillus amyloliquefaciens* from the difficidin (Dfn), bacillaene (Bae), and bacillomycin D (Bmy) biosynthesis pathways. These observations may suggest that some, or possibly all, of the *T. turnerae* secondary metabolite regions may have originated from lateral gene transfer events.

### Bacteriophage, restriction-modification and mobile-DNA

The intracellular environment of *T. turnerae* is thought to provide it limited exposure to mobile genetic elements. Nonetheless, the genome of *T. turnerae* indicates a history of exposure to foreign genetic material, including bacteriophage (Supporting Information: Table S5) and transposable elements. Two gene clusters in the *T. turnerae* genome, *cas/csd* and *cmr*, are predicted to function in generating and maintaining clusters of regularly interspaced short palindromic repeats (CRISPRs) (Supporting Information: Figure S3A). CRISPR and associated genes provide bacteria with acquired immunity against infection by bacteriophage, and possibly other extrachromosomal elements [80], via an incompletely understood mechanism involving incorporation of short non-coding and non-repetitive spacer sequences derived from phage during viral challenge [81].

Based on the arrangement, orientation and sequence of CRISPR-associated genes, including Cas5 (TERTU_3118), it is likely that *T. turnerae* encodes the Dvulg subtype of the Cas guild [82]. Thirty-one units of direct repeats, each 32 bp in length, were identified downstream of the *cas2* gene. Each of these CRISPR repeats is capable of forming a hairpin structure when transcribed, consisting of a 7 base-pair stem and a loop of 5 nucleotides (Supporting Information: Figure S3B). As has been described for other CRISPR bacterial systems, the terminal repeat sequence is a variant of other repeats in the unit [81]. Additionally, the secondary structure of the *T. turnerae* repeats is consistent with the cluster 3 type of CRISPRs, which are also associated with Dvulg Cas genes [83]. CRISPR and associated genes have been identified in the *C. japonicus* genome [16] but not in *S. degradans*.

The *T. turnerae* genome encodes 30 CRISPR spacers with a mean length of 34 nucleotides. Two of these spacers closely matched (20 of 22 base pairs) regions of the *Vibrio* phage VHML encoding a putative baseplate spike protein (orf30) and the *Ralstonia* phage phiRSA1 encoding an endonuclease subunit (ORF9) respectively, however, exact cognate bacteriophage sequences were not identified in public databases.

DNA restriction-modification (R-M) systems have also been proposed as a mechanism of bacteriophage immunity. While it remains challenging to identify endonuclease (R) genes bioinformatically, nucleic acid methylase (M) genes are well conserved. There are at least two R-M systems in the *T. turnerae* genome anchored by methylase genes TERTU_3913 and TERTU_2390. Both are predicted to be type I R-M systems, and as such are associated with genes that encode specificity (S) subunits. In comparison, two type I R-M systems and two type III methylases were identified in *C. japonicus* (data not shown). No R-M associated methylase genes were identified in *S. degradans*.

### Conclusion

Detailed genomic information is available for comparatively few intracellular endosymbionts. Therefore, a central question with regard to each of these genomes is to what extent does genome content and organization reflect adaptation to this ecologically important niche. Unlike most intracellular symbionts examined to date, *T. turnerae* is capable of growth *in vitro* under simple defined conditions. However, despite considerable effort, this bacterium has never been isolated from any environment other than the gill tissue of teredinid bivalves. Thus, although *T. turnerae* appears to be capable of independent existence, the extent to which this bacterium may grow and/or reproduce outside of its host in nature remains unknown.

Genome content and organization may provide evidence of the extent to which *T. turnerae* is restricted to the intracellular environment. Considerable attention has been paid to intracellular pathogens and endosymbionts of animals and the broad syndrome of genomic modifications associated with increasing dependence on intracellular existence. Such modifications may include reduction in genome size, decreased %G+C, increased fixation of harmful mutations, loss of genes of core metabolism including DNA repair, formation of pseudogenes, transitory proliferation of insertion elements, and reduction in number of ribosomal operons and tRNA genes [9], [47], [84].

Contrary to observations on the genomes of other bacteria that form stable, long-term, intracellular associations with animals, the circular genome of *T. turnerae* is comparatively large. In fact, it is larger and contains significantly more predicted open reading frames than has been reported for its closest known free-living relative, *S. degradans* (Table 1). Also, unlike other intracellular symbionts, the genome of *T. turnerae* shows no reduction in %G+C compared to its closest free-living relative but is in fact 5% greater. It contains a greater number of ribosomal RNA genes and a comparable number of tRNA genes. Moreover, *T. turnerae* maintains a complete complement of genes involved in virtually all core metabolic functions including DNA repair and contains only three predicted pseudogenes as compared to nine in *S. degradans*. Although the number of insertion elements detected in *T. turnerae* significantly exceeds that of *S. degradans*, this number is consistent with the profiles of pathogens, such as *Listeria monocytogenes* [85], and is far less than that determined for recently examined facultative endosymbionts [86]. Also contrary to expectations for an intracellular endosymbiont, the genome of *T. turnerae* suggests a recent history of exposure to bacteriophage. The *T. turnerae* genome encodes and maintains a phage defense arsenal that includes CRISPR and at least two restriction modification systems and contains a number of phage elements similar to that of its free-living relative, *S. degradans*.

Another interesting feature of the *T. turnerae* genome is the magnitude of its secondary metabolic potential. A proposed function of secondary metabolites is to act as antimicrobials to suppress competition from other bacteria. This again could suggest recent or current competition with free-living bacteria. However, secondary metabolites might also play a role in symbiosis. It should be noted that *T. turnerae* is closely related to *Candidatus Endobugula sertula*, a bacterial symbiont of bryozoans. This symbiont is proposed to be responsible for the biosynthesis of bryostatins, polyketides that protect the bryozoan larvae from predation. The metabolites of *T. turnerae* may play a similar role in the shipworm symbiosis. Alternatively, they could suppress microbial competitors for infection of the host tissues or digestive system, defend the host against pathogens, or play a role in communication with the host. Finally, shipworm gills typically contain several related bacterial species in addition to *T. turnerae*. Thus secondary metabolites may be important in community assembly within the gill tissue by regulating competing populations of coexisting endosymbionts.

In summary, the genome of *T. turnerae* suggests that of a facultative endosymbiont that either maintains a significant ecological niche outside of its host, or of a bacterium that has only recently become restricted to the intracellular environment. We can identify no feature, or combination of features, of its genome content or organization that uniquely identifies *T. turnerae* as a symbiotic bacterium. Nor can we identify, based on bioinformatics, any gene that can uniquely be identified as a “symbiosis gene”, although there is some indication that such genes may exist. Given the widespread occurrence and prevalence of *T. turnerae* among phylogenetically and biogeographically diverse shipworm taxa, and the long fossil history of these host groups, we favor the hypothesis that *T. turnerae* has maintained a long history of relatively independent facultative association with shipworms. It remains unclear why the evolutionary trajectory of this symbiosis has not lead to evidence of greater host dependence. Additional, comparative genomic analysis of cultivated and as yet uncultivated shipworm symbiont strains and species may lead to a better understanding of this elusive question.

## Materials and Methods

### Provenance of the sequenced strain

The sequenced strain *T. turnerae* T7901 was isolated by John Waterbury, Woods Hole Oceanographic Institution from a specimen of the shipworm *Bankia gouldi* collected from the Newport River Estuary, Beaufort North Carolina in 1979. This strain, and 53 similar strains of *T. turnerae* isolated from a variety of shipworm species by Waterbury *et al*. [1], [2] between 1979 and 1986, have been deposited to the Ocean Genome Resource, (OGR accession number I00002) a public biorepository operated by Ocean Genome Legacy, Inc. The sequenced strain has also been deposited to the American Type Culture Collection (accession number 39867).

### Cultivation of Teredinibacter turnerae

Strains were grown in liquid batch culture in shipworm basal medium (SBM) as previously described [1]. SBM was supplemented with sucrose (final concentration 0.5%) and ammonium chloride (final concentration 5 mM). Difco agar (1%) was added for plate cultures. For genomic DNA extractions, a single colony of *T. turnerae* was used to inoculate 100 ml aliquots of SBM. Cultures were incubated with mild agitation (100 rpm) at 30°C until optical densities between 0.08 and 0.10 OD600 units were achieved. The resulting cell pellet was harvested by centrifugation at 12,000 rpm for 15 minutes.

### Genomic DNA preparation

A cell pellet of approximately 125 µl in volume was resuspended in 4.75 ml of TE 8.0 buffer (10 mM Tris, pH 8.0, 1 mM EDTA). After addition of 1.25 ml of 10% SDS and 12.5 µl of Proteinase K (20%), the suspension was mixed by inversion and incubated at 37°C for 60 minutes. To this suspension was added 600 µl of NaCl (5 M) and 375 µl CTAB (10% in 0.7 M NaCl) prewarmed to 65°C. After incubation at 65°C for 20 min., the suspension was allowed to cool to room temperature, 6 ml of dichloromethane was added. The suspension was then mixed by inversion, phases were separated by centrifugations (8000×g, 15 min), and the aqueous phase was retained and subjected to 2 additional rounds of extraction with dichloromethane. Nucleic acids were precipitated from the solution by addition of 0.65 volumes of isopropanol, spooled on a glass rod, washed by submersion in EtOH (100%), dissolved in 500 µl of TE 8.0 buffer containing 1 µl RNase (100 mg/ml), and incubated at 37°C for 60 min. After addition of NaOAc (0.3 M final concentration), two volumes of EtOH (100%), were added and the DNA was spooled onto a glass rod, washed with EtOH (100%) and dissolved in 500 µl of TE 8.0 buffer. Genomic DNA was subsequently purified using DNeasy mini spin columns (Qiagen) according to the manufacturer's recommended protocol.

### Genome sequencing and assembly

The complete genome sequence was determined using a combination of Sanger [87] and Roche-454 GS20 [88] technologies as described in [89]. Three libraries were made – a small insert library of 3 to 4 Kb, a medium insert library of 8 to 10 Kb, and a fosmid library with inserts of 33–39 Kb. A total of 6144 Sanger reads were performed for each library. A single plate of 454 GS20 sequencing (158,697 reads) was then performed to supplement the Sanger sequence. The Celera Assembler [90] and JCVI's in-house hybrid assembly method were used to assemble the combined Sanger/454 data, resulting in three sequence scaffolds containing 28 intra-scaffold gaps. The total number of sequence gaps was reduced to 13 using JCVI's automated closure procedure (Hostetler et al., unpublished) which analyzes assembly results, identifies finishing targets, designs primers, selects clones, and chooses and performs sequencing reactions in an automated pipeline. The remaining sequence gaps and low sequence coverage areas were resolved manually using a combination of primer walking, PCR and transposon-mediated sequencing. The jump-start feature of the Celera Assembler was used for final sequence assembly achieving a final coverage of 21.2X.

### Sequence annotation

An initial set of predicted protein-coding regions was identified using GLIMMER [91], [92]. Those shorter than 30 amino acids and those with overlaps of higher scoring regions were eliminated. The likely origin of replication was identified and base pair 1 was designated adjacent to the dnaA gene [93]. Putative protein functions were assigned using JCVI's AutoAnnotate pipeline which searches against an in-house non-redundant protein database using BLASTP [94] then extends the BLASTP results using the BLAST-Extend-Repraze (BER) method to improve identification of gene boundaries. Putative proteins were also compared to two sets of hidden Markov models (HMMs): Pfam HMMs [95], and TIGRFAMs [96] using the HMMER package [97]. HMMs were built from highly curated multiple alignments of proteins thought to share the same function or to be members of the same protein family. Approximately 70% of putative proteins whose functions were predicted by auto-annotation were subjected to individual manual inspection and “expert” annotation by the authors. The complete genome sequence has submitted to GenBank (accession # CP001614).

### Secretome analysis

Protein expression/secretion was examined in *T. turnerae* grown with three different carbon sources. After two days of growth in liquid medium containing either sucrose, carboxymethylcellulose (CMC, a soluble form of cellulose), or SigmaCell (SMC, insoluble cellulose powder) as the sole carbon source, spent *Teredinibacter turnerae* fermentation medium was cleared of cells by centrifugation and used for proteomic analysis. An 800 µL aliquot of each cleared fermentation medium was injected onto an 1100/1200 Series Liquid Chromatography System (Agilent Technologies) and separated on a PLRP-S reversed-phase column (1 mm×150 mm; Higgins Analytical, Inc.) using a 45 min 15–60% TB gradient (TA = 0.1% trifluoroacetic acid, TB = CH_3_CN, 0.1% trifluoroacetic acid) at a flow rate of 100 µL min^−1^. Fractions containing protein were identified by UV (214 nm) and the intensity of absorbance at this wavelength was used to determine the protein concentration in each fraction. Fractions were individually dried to completion under vacuum. Proteins from each dried fraction were resuspended in 30 µL Trypsin reaction buffer (50 mM Tris-HCl, 20 mM CaCl_2_, pH 8) and digested overnight at 37°C with 100 ng of Trypsin (New England Biolabs, Inc.). Eight µL of each digested fraction was injected into a HPLC-Chip Cube system and separated on a Protein ID chip comprised of a 40 nL enrichment column, a 75 µm×150 mm separation column packed with Zorbax 300SB-C18 5 µm material, and a spray emitter with a 15 µm flow path (Agilent Technologies). Peptides were separated using a 40 min 5–45% FB linear gradient (FA = 0.1% formic acid, FB = CH_3_CN, 0.1% formic acid) at a flow rate of 0.4 µL min^−1^ and analyzed online by a 6330 Ion Trap Mass Spectrometer with a Nano-Electrospray (nanoESI) ionization source (Agilent Technologies). A capillary voltage of 1700–1900 V (optimized on a per-chip basis) was used and the skimmer voltage was held at 30 V. Data were acquired at 25,000 m/z·sec^−1^ with a SmartTarget value of 500,000 and Maximum Accumulation Time of 200 ms. The MS acquisition range was from 300 to 1800 m/z. Default parameters for Auto MS^2^ were used. Ions were selected for fragmentation based on their intensity, with the number of precursor ions set to 5. The MS/MS Fragmentation Amplitude was set to 1.30 V with SmartFrag Start and End Amplitude values set to 30 and 200%, respectively. The MS/MS acquisition range was from 50 to 2200 m/z. Data acquisition was coordinated with the start of the LC separation and was stopped after 60 min. Protein separation, digestion and peptide analysis were repeated in triplicate.

The ESI-MS/MS data were analyzed using both Spectrum Mill (Agilent Technologies) and Mascot (Version 2.2, Matrix Science Ltd.) search engines [98]. For the Spectrum Mill analysis, the raw data were processed by Spectrum Mill MS Proteomics Workbench (Rev A.03.02.060b). The default settings in Data Extractor were used to prepare MS/MS data files for Spectrum Mill processing. Processed data were then subjected to an MS/MS Search using a *T. turnerae* database. The search criteria were set to allow two missed cleavages by trypsin with no protein modifications. The precursor mass tolerance and product mass tolerance were set to±2.5 and±0.7 Da, respectively. Peptides were filtered by a Score >7 and a % SPI>60 and only proteins scoring better than 20 were considered valid identifications. For the Mascot analysis, raw data were converted to .mgf files by DataAnalysis (Version 6.1, Agilent Technologies). Compounds were detected using the following method parameters: S/N threshold set to 2, Intensity threshold set to 100 for both positive and negative, and a maximum number of 8000 compounds with a retention time window of 0.05 min. The .mgf files were uploaded to Mascot and searched against a *T. turnerae* database. The search criteria were set to allow two missed cleavages by a semi-trypsin digest with no protein modifications. The tolerances for peptide and MS/MS were set to 1.2 and 0.6 Da, respectively. Peptide charges of 1+, 2+ and 3+ were selected with MudPIT scoring and an “ion score or expect cut-off” value of 20. Proteins identified with a Probability Based Mowse score of 67 or better were considered valid identifications (p<0.05).

## Supporting Information

Table S1 Glycoside hydrolases of *T. turnerae* (99 ORFs total; 101 domains total).
(0.15 MB DOC)

Table S2 Polysaccharide lyases of *T. turnerae* (4 ORFs total; 5 domains total).
(0.04 MB DOC)

Table S3 Carbohydrate esterases of *T. turnerae* (22 ORFs total; 22 domains total).
(0.06 MB DOC)

Table S4 Carbohydrate binding domain encoding ORFs not associated with GH, PL and CE domains in *T. turnerae* (17 ORFs total, 24 domains total). PFAM-A abbreviations are used for non-CBM domains.
(0.05 MB DOC)

Table S5 Prophage associated genes in *T. turnerae*.
(0.04 MB DOC)

Figure S1 Circular representation of the chromosome of *T. turnerae* T7901. Circular plots in order from outermost to innermost rings: 1) and 2) predicted protein coding regions (blue), tRNA genes (red), and rRNA genes (pink) in the forward and reverse strands respectively, 3) local G+C content of the genome (black), with high and low G+C regions represented by peaks facing away from or toward the center of the figure respectively, 4) GC-skew (positive values in green, negative in pink), and 5) distance in base pairs from the predicted origin of replication. Note that changes in the sign of GC skew correspond with and support the predicted origin and terminus of replication.
(2.15 MB TIF)

Figure S2 Nit1C gene cluster organization. The genomic context of the *T. turnerae* nitrilase gene (TERTU_04234) is shown along with similar nitrilase operons (Nit1C) from other bacterial genomes. Other proteins encoded by genes commonly found in Nit1C clusters include 2 hypothetical proteins (hyp1 and hyp2), a radical SAM superfamily protein (SAM, Pfam 04055), GCN-5 related acetyltransferse (GNAT, Pfam 00583), 5′-phosphorybosyl-5-aminoimidazole synthase-related proteins (AIRS, Pfam 00586), and a putative flavoprotein (Flavo).
(0.29 MB TIF)

Figure S3 CRISPR associated genetic loci in *Teredinibacter turnerae*. The CRISPR associated cas/csd and cmr loci are shown (A). Genes belonging to different gene families are distinguished by color (blue, cas; red, csd; purple, cmr). The predicted CRISPR repeat RNA hairpin structure is shown (green) with the variant terminal repeat (orange). Hairpin sequences are oriented with respect to the cas operon, which is antisense to the genome.
(0.19 MB TIF)
