# Supplementary material for: The Complete Genome of Teredinibacter turnerae T7901: An Intracellular Endosymbiont of Marine Wood-Boring Bivalves (Shipworms)
Source: PLoS One. 2009 Jul 1;4(7):e6085. doi: 10.1371/journal.pone.0006085 (PMC2699552; doi:10.1371/journal.pone.0006085)
Supplement: Table S1 — Glycoside hydrolases of T. turnerae (99 ORFs total; 101 domains total). (0.15 MB DOC) [file pone.0006085.s001.doc]

Supporting Information: Table SI. Glycoside hydrolases of *T. turnerae* (99 ORFs total; 101 domains total).

| **ORF^a^** | **Predicted Function** | **Modular Architecture *** | **PolyS** | **SignalP** | **LipoP** |
| --- | --- | --- | --- | --- | --- |
| TERTU_3167 | sucrose phosphorylase | GH13 | no | no | no |
| TERTU_3210 | beta-glucosidase | GH3 | no | no | no |
| TERTU_3253 | beta-xylosidase | GH52 | no | no | no |
| TERTU_3256 | beta-glycosidase | GH16-CBM10-CBM5 | yes | yes | no |
| TERTU_3341 | sucrose phosphorylase | GH13 | no | no | no |
| TERTU_3347 | xyloglucanase | GH74-CBM10-CBM2 | yes | yes | no |
| TERTU_3349 | xylanase | GH10 | yes | yes | yes |
| TERTU_3361 | beta-glucanase | CBM2-CBM10-GH5 | yes | yes | no |
| TERTU_3398 | xylanase | GH10 | no | yes | no |
| TERTU_3400 | endoglucanase | CBM2-CBM10-GH45 | yes | yes | no |
| TERTU_2843 | alpha-glucuronidase | GH67 | no | yes | no |
| TERTU_2842 | beta-glucosidase | GH3 | no | no | yes |
| TERTU_2767 | beta-glycan phosphorylase | GH94 | no | no | no |
| TERTU_2762 | cellobiose phosphorylase | GH94 | no | no | no |
| TERTU_1233 | muramidase | GH73 | no | no | no |
| TERTU_0676 | beta-galactosidase | GH2 | no | yes | yes |
| TERTU_0645 | endoglucanase | GH9-CBM10-CBM2 | yes | yes | no |
| TERTU_0607 | endoglucanase | GH9 | no | yes | yes |
| TERTU_0605 | peptidoglycan lytic transglycosylase | GH103 | no | yes | no |
| TERTU_0513 | xylanase | GH10-CBM22-CBM22-CBM22-CBM22-CBM22 | no | yes | no |
| TERTU_0428 | xylanase and cellodextrinase | GH11-CBM5-CBM10-GH5 | yes | yes | no |
| TERTU_0427 | cellodextrinase | CBM5-CBM10-GH5 | yes | yes | no |
| TERTU_0353 | xyloglucanases | GH12 | no | yes | no |
| TERTU_0285 | chitinase | CBM5-CBM5-GH18 | no | no | no |
| TERTU_0183 | endoglucanase | GH5 | no | yes | no |
| TERTU_0149 | beta-mannanase | CBM2-CBM10-CBM10-CBM5-GH26 | yes | yes | no |
| TERTU_4701 | beta-glycosidase | GH16<CBM32>-CBM32-CBM32 | no | yes | no |
| TERTU_4678 | xylosidase/arabinofuranosidase | GH43 | no | yes | yes |
| TERTU_4676 | xylanase | GH10 | no | yes | yes |
| TERTU_4675 | xylosidase/arabinofuranosidase | GH43 | no | yes | yes |
| TERTU_4506 | xylanase | GH8 | no | yes | yes |
| TERTU_4415 | beta-mannosidase | GH5 | no | yes | yes |
| TERTU_4345 | beta-glycosidase | GH9 | no | yes | no |
| TERTU_4344 | beta-mannanase | GH5-CBM10-CBM10-CBM10 | yes | yes | no |
| TERTU_4269 | xylanase | CBM10-GH5 | yes | no | no |
| TERTU_4238 | putative alpha-L-arabinofuranosidases | GH62 | no | yes | yes |
| TERTU_4225 | alpha-L-arabinofuranosidase | CBM2-CBM6-GH62 | yes | yes | no |
| TERTU_4222 | xylosidase/arabinofuranosidase | GH43 | no | yes | yes |
| TERTU_4094 | beta-mannanase | CBM35-GH26 | no | yes | yes |
| TERTU_4091 | alpha-galactosidase | GH27 | no | yes | no |
| TERTU_4090 | beta-glycosidase | GH5-FN3 | no | no | no |
| TERTU_4086 | beta-glucosidase | GH3 | no | yes | yes |
| TERTU_4085 | beta-glycosidase | GH5 -CBM6 | no | yes | no |
| TERTU_4083 | beta-1,3-glucanase | GH16-CBM6-CBM32-CBM32 | no | yes | no |
| TERTU_4054 | endoglucanase | GH44 | no | yes | no |
| TERTU_4019 | endoglucanase | GH9-CBM2-CBM13 | yes | yes | no |
| TERTU_3996 | [non-reducing end] cellobiohydrolase | GH6 | yes | yes | yes |
| TERTU_3990 | putative N-acetylmuramidases | GH108 | no | no | no |
| TERTU_3987 | beta-1,3-glucanase | CBM2-CBM13-GH16 | no | yes | no |
| TERTU_3950 | putative peptidoglycan hydrolases | GH73 | no | yes | no |
| TERTU_3751 | beta-glycosidase | GH5 | no | yes | no |
| TERTU_3750 | beta-glucosidase | GH3 | no | no | no |
| TERTU_3690 | beta-glycosidase | GH5 | no | yes | yes |
| TERTU_3605 | alpha-glycosidase | GH15 | no | no | no |
| TERTU_3603a | acetylxylan esterase and xylanase | CE6-CBM5-CBM10-GH10 | yes | yes | no |
| TERTU_3565 | endoglucanase | CBM2-GH5 | yes | yes | no |
| TERTU_3487 | beta-glycosidase | GH79 | no | no | no |
| TERTU_3447a | xylanase and methylglucuronoyl esterase | GH11-CBM5-CE15 | yes | no | no |
| TERTU_2894 | beta-mannanase | GH5 -CBM2-CBM10-CBM10 | yes | yes | no |
| TERTU_2893 | endoglucanase | GH9-CBM3-CBM5-CBM10 | yes | yes | no |
| TERTU_2595 | lytic murein transglycosylase | GH23 | no | no | no |
| TERTU_2546 | xylanase | CBM2-CBM10-GH10 | yes | yes | no |
| TERTU_2458 | lytic murein transglycosylases | GH23 | no | yes | no |
| TERTU_2329 | xylosidase/arabinofuranosidase | GH43 | no | yes | no |
| TERTU_2313 | beta-xylosidase | GH39 | no | yes | no |
| TERTU_2311 | endoglucanase | GH44-CBM5-CBM2 | yes | yes | no |
| TERTU_2309 | endo-1,4-beta-galactanases | CBM10-CBM10-GH53 | yes | yes | no |
| TERTU_2298 | beta-xylosidase | GH39 | no | no | no |
| TERTU_2211 | beta-glycosidase | CBM2-CBM10-GH16 | yes | yes | no |
| TERTU_2108 | lytic murein transglycosylase | CBM50-CBM50-CBM50-GH23 | no | no | yes |
| TERTU_1941 | beta-glucosidase | GH3 | no | no | no |
| TERTU_1883 | putative lytic murein transglycosylases | GH23 | no | yes | yes |
| TERTU_1681 | xylanase | GH11 | no | yes | no |
| TERTU_1680a | xylanase and acetylxylan esterase | GH11-CE4-CBM10 | yes | yes | no |
| TERTU_1678a | xylanase and acetylxylan esterase | GH11-CE4-CBM10 | yes | yes | no |
| TERTU_1675 | beta-1,3-glucanase | GH16-CBM6* | no | yes | no |
| TERTU_1599 | xylanase | GH10-CBM6-CBM22-CBM22 | no | yes | no |
| TERTU_1500 | alpha-L-fucosidase | GH95 | no | yes | no |
| TERTU_1499 | beta-galactosidase | GH35 | no | yes | no |
| TERTU_1498 | alpha-glycosidase | GH31 | no | yes | yes |
| TERTU_1491 | polygalacturonase | GH28 | no | yes | yes |
| TERTU_1488 | unsaturated uronyl hydrolases | GH105 | no | yes | yes |
| TERTU_1485 | xylosidase/arabinofuranosidase | CBM13-CBM35-GH43 | no | yes | no |
| TERTU_1419 | xylosidase/arabinofuranosidase | GH43 | no | no | no |
| TERTU_0937 | glycogen debranching enzyme | GH13 | no | no | no |
| TERTU_0936 | 1,4-alpha-glucan branching enzyme | GH13 | no | no | no |
| TERTU_0935 | 4-alpha-glucanotransferase | GH77 | no | no | no |
| TERTU_0856 | lytic murein transglycosylases | GH23 | no | yes | no |
| TERTU_0851 | beta-glycan phosphorylase | GH94 | no | no | no |
| TERTU_0835 | xylanase | GH10 | yes | yes | yes |
| TERTU_0817 | related to xylanases | GH10 | no | no | no |
| TERTU_0811 | N-acetyl-beta-glucosaminidase | GH20 | no | yes | no |
| TERTU_0782 | beta-glycosidase | GH9 | no | yes | no |
| TERTU_0768 | alpha-L-arabinofuranosidase | GH51 | no | yes | no |
| TERTU_0767 | endoglucanase | GH45 | yes | yes | yes |
| TERTU_0763 | xylanase | GH5 | yes | yes | yes |
| TERTU_0736 | xylanase | GH10 | no | yes | no |
| TERTU_2895a | endo- and exoglucanase, celA | GH5-CBM5-CBM10-GH6 | yes | yes | no |
| TERTU_2898 | cellobiohydrolase | CBM5-CBM10-GH6 | yes | yes | no |

a. ORF encodes multiple catalytic domains

* partial/truncated domain

<> embedded domain
