# Supplementary material for: The Complete Genome of Teredinibacter turnerae T7901: An Intracellular Endosymbiont of Marine Wood-Boring Bivalves (Shipworms)
Source: PLoS One. 2009 Jul 1;4(7):e6085. doi: 10.1371/journal.pone.0006085 (PMC2699552; doi:10.1371/journal.pone.0006085)
Supplement: Table S2 — Polysaccharide lyases of T. turnerae (4 ORFs total; 5 domains total). (0.04 MB DOC) [file pone.0006085.s002.doc]

Supporting Information: Table S2. Polysaccharide lyases of *T. turnerae* (4 ORFs total; 5 domains total).

| **ORF^a^** | **Predicted Function** | **Modular Architecture*** | **PolyS** | **SignalP** | **LipoP** |
| --- | --- | --- | --- | --- | --- |
| TERTU_3356 | rhamnogalacturonan lyase | PL11-CBM6-CBM2 | yes | yes | no |
| TERTU_3387 | pectate lyase | CBM2*-CBM10-PL11 | yes | no | no |
| TERTU_0130 | polysaccharide lyase | PL1 | yes | no | no |
| TERTU_1483a | pectate lyase | PL1-CBM32-CBM5-CBM35-PL1 | yes | yes | no |

a. ORF encodes multiple catalytic domains

* partial/truncated domain
