# Supplementary material for: The Complete Genome of Teredinibacter turnerae T7901: An Intracellular Endosymbiont of Marine Wood-Boring Bivalves (Shipworms)
Source: PLoS One. 2009 Jul 1;4(7):e6085. doi: 10.1371/journal.pone.0006085 (PMC2699552; doi:10.1371/journal.pone.0006085)
Supplement: Table S3 — Carbohydrate esterases of T. turnerae (22 ORFs total; 22 domains total). (0.06 MB DOC) [file pone.0006085.s003.doc]

Supporting Information: Table S3. Carbohydrate esterases of *T. turnerae* (22 ORFs total; 22 domains total).

| **ORF^a^** | **Predicted Function** | **Modular Architecture** | **PolyS** | **SignalP** | **LipoP** |
| --- | --- | --- | --- | --- | --- |
| TERTU_2703 | esterase | CE3-CBM10-CBM2 | yes | yes | no |
| TERTU_0657 | esterase | CE4 | no | yes | no |
| TERTU_0517 | methylglucuronoyl esterases | CE15 | no | yes | no |
| TERTU_0453 | feruloylesterase | CE1 | no | no | no |
| TERTU_0446 | esterase | CE1 | yes | yes | no |
| TERTU_0094 | esterase | CE1 | no | yes | no |
| TERTU_4339 | S-formylglutathione transferase | CE1 | no | no | no |
| TERTU_4299 | esterase | CE1 | no | yes | no |
| TERTU_4241 | esterase | CE12 | no | yes | no |
| TERTU_3603a | acetylxylan esterase and xylanase | CE6-CBM5-CBM10-GH10 | yes | yes | no |
| TERTU_3514 | methylglucuronoyl esterase | CE15 | yes | yes | yes |
| TERTU_3447a | xylanase and methylglucuronoyl esterase | GH11-CBM5-CE15 | yes | no | no |
| TERTU_3043 | N-acetylglucosamine deacetylase | CE11 | no | no | no |
| TERTU_2321 | esterase | CBM2-CBM35-CE12 | yes | yes | no |
| TERTU_2320 | esterase | CBM2-CBM35-CBM6-CE12 | yes | no | no |
| TERTU_2314 | pectin methylesterase | CBM2-CBM35-CBM6-CE8 | no | yes | no |
| TERTU_2014 | polysaccharide deacetylase | CE4 | no | no | no |
| TERTU_1680a | xylanase and acetylxylan esterase | GH11-CE4-CBM10 | yes | yes | no |
| TERTU_1678a | xylanase and acetylxylan esterase | GH11-CE4-CBM10 | yes | yes | no |
| TERTU_1484 | pectin methylesterase | CE8 | no | no | no |
| TERTU_0847 | esterase | CE2 | no | yes | no |
| TERTU_0814 | N-acetylglucosamine 6-phosphate deacetylase | CE9 | no | no | no |

a. ORF encodes multiple catalytic domains
