# Supplementary material for: The Complete Genome of Teredinibacter turnerae T7901: An Intracellular Endosymbiont of Marine Wood-Boring Bivalves (Shipworms)
Source: PLoS One. 2009 Jul 1;4(7):e6085. doi: 10.1371/journal.pone.0006085 (PMC2699552; doi:10.1371/journal.pone.0006085)
Supplement: Table S4 — Carbohydrate binding domain encoding ORFs not associated with GH, PL and CE domains in T. turnerae (17 ORFs total, 24 domains total). PFAM-A abbreviations are used for non-CBM domains. (0.05 MB DOC) [file pone.0006085.s004.doc]

Supporting Information: Table S4. Carbohydrate binding domain encoding ORFs not associated with GH, PL and CE domains in *T. turnerae* (17 ORFs total, 24 domains total). PFAM-A abbreviations are used for non-CBM domains.

| **ORF** | **Predicted Function** | **Modular Architecture** | **PolyS** | **SignalP** | **LipoP** |
| --- | --- | --- | --- | --- | --- |
| TERTU_2699 | peptidase | peptidase_S8-CBM2 | no | yes | no |
| TERTU_0650 | alpha-glucan-binding protein | CBM48 | no | no | no |
| TERTU_0468 | carbohydrate binding domain protein | CBM32-DUF1111 | no | yes | no |
| TERTU_0331 | carbohydrate binding domain protein | CBM2 | no | yes | no |
| TERTU_0129 | carbohydrate-binding protein | CBM13 | no | yes | no |
| TERTU_0047 | carbohydrate binding domain protein | CBM2 | no | yes | no |
| TERTU_0046 | chitin-binding protein | CBM33-CBM10 | yes | yes | no |
| TERTU_4665 | TonB-dependent receptor | Plug-CBM4 | no | yes | no |
| TERTU_4597 | carbohydrate binding domain protein | CBM10-CBM10-CBM10-CBM10 | no | yes | no |
| TERTU_4541 | carbohydrate binding domain protein | CBM2 | no | yes | no |
| TERTU_3803 | gluconolactonase | CBM10-SGL-CBM2-PSD5-PSD4-PSCyt3 | yes | yes | no |
| TERTU_3477 | carbohydrate binding domain protein | CBM4 | no | no | no |
| TERTU_2684 | carbohydrate binding domain protein | CBM2-CBM35 | no | yes | no |
| TERTU_2567 | glucose/sorbosone dehydrogenase | GSDH-PA14-He_PIG-CBM10-CBM5 | yes | yes | no |
| TERTU_2195 | serine protease | peptidase_S8-CBM5 | no | yes | no |
| TERTU_1900 | carbohydrate binding domain protein | CBM9 | no | yes | no |
| TERTU_1674 | carbohydrate binding domain protein | CBM6 | no | no | no |
