# Supplementary material for: The Complete Genome of Teredinibacter turnerae T7901: An Intracellular Endosymbiont of Marine Wood-Boring Bivalves (Shipworms)
Source: PLoS One. 2009 Jul 1;4(7):e6085. doi: 10.1371/journal.pone.0006085 (PMC2699552; doi:10.1371/journal.pone.0006085)
Supplement: Table S5 — Prophage associated genes in T. turnerae. (0.04 MB DOC) [file pone.0006085.s005.doc]

Supporting Information: Table S5. Prophage associated genes in *T. turnerae*

| **gene** | **predicted gene function** |
| --- | --- |
| TERTU_0478 | phage protein Gp37/Gp68 |
| TERTU_1424 | site-specific recombinase, phage integrase family |
| TERTU_1429 | bacteriophage replication gene A protein |
| TERTU_1976 | site-specific recombinase, phage integrase family |
| TERTU_2220 | conserved hypothetical phage tail protein |
| TERTU_2227 | phage GpW/Gp25 family protein |
| TERTU_2261 | site-specific recombinase, phage integrase family |
| TERTU_3339 | site-specific recombinase, phage integrase family |
| TERTU_3502 | phage tail collar domain protein |
