# Supplementary figures and images for: The Complete Genome of Teredinibacter turnerae T7901: An Intracellular Endosymbiont of Marine Wood-Boring Bivalves (Shipworms)
Source: PLoS One. 2009 Jul 1;4(7):e6085. doi: 10.1371/journal.pone.0006085 (PMC2699552; doi:10.1371/journal.pone.0006085)

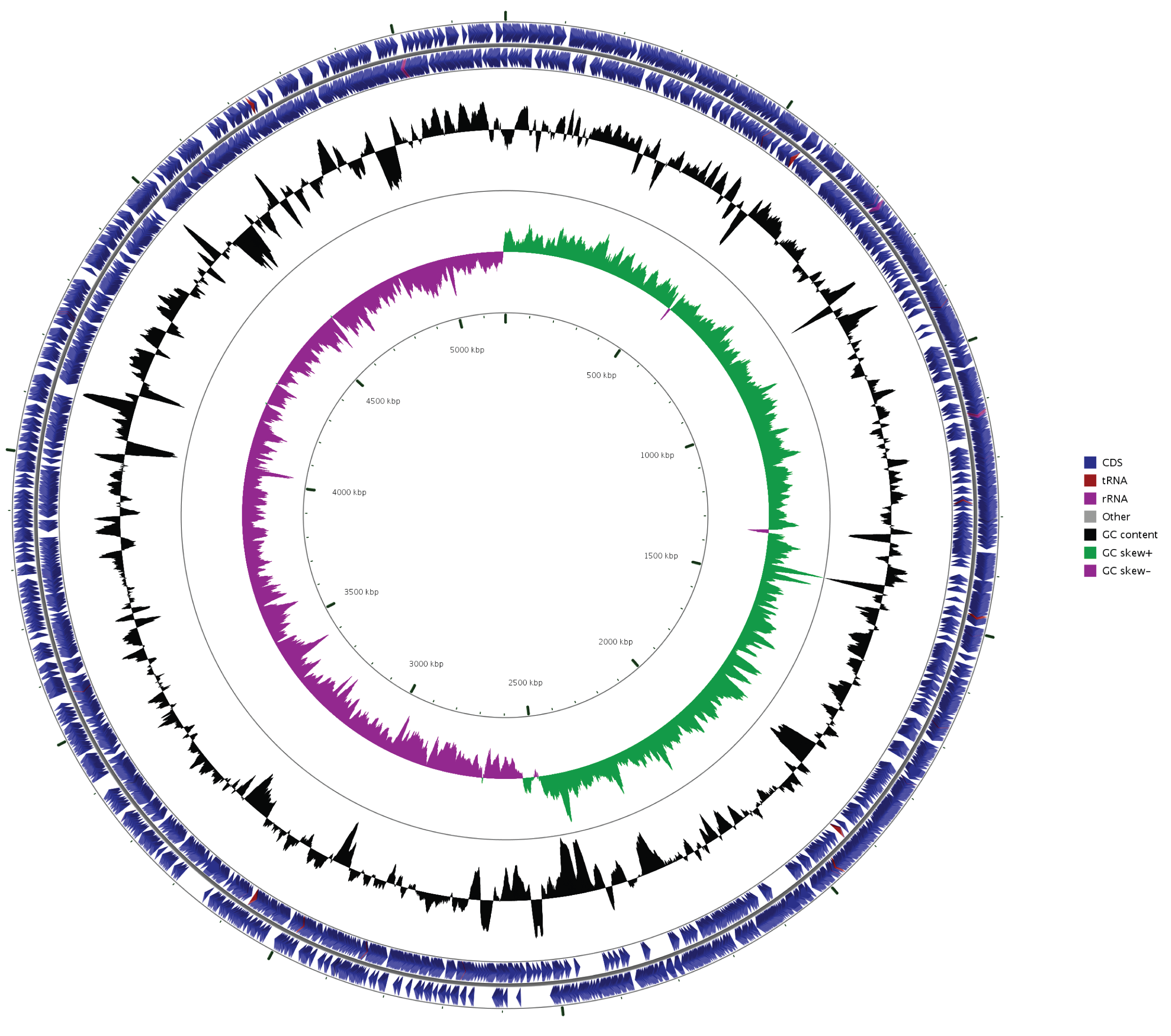

Supplement: Figure S1 — Circular representation of the chromosome of T. turnerae T7901. Circular plots in order from outermost to innermost rings: 1) and 2) predicted protein coding regions (blue), tRNA genes (red), and rRNA genes (pink) in the forward and reverse strands respectively, 3) local G+C content of the genome (black), with high and low G+C regions represented by peaks facing away from or toward the center of the figure respectively, 4) GC-skew (positive values in green, negative in pink), and 5) distance in base pairs from the predicted origin of replication. Note that changes in the sign of GC skew correspond with and support the predicted origin and terminus of replication. (2.15 MB TIF) [file pone.0006085.s006.tif]

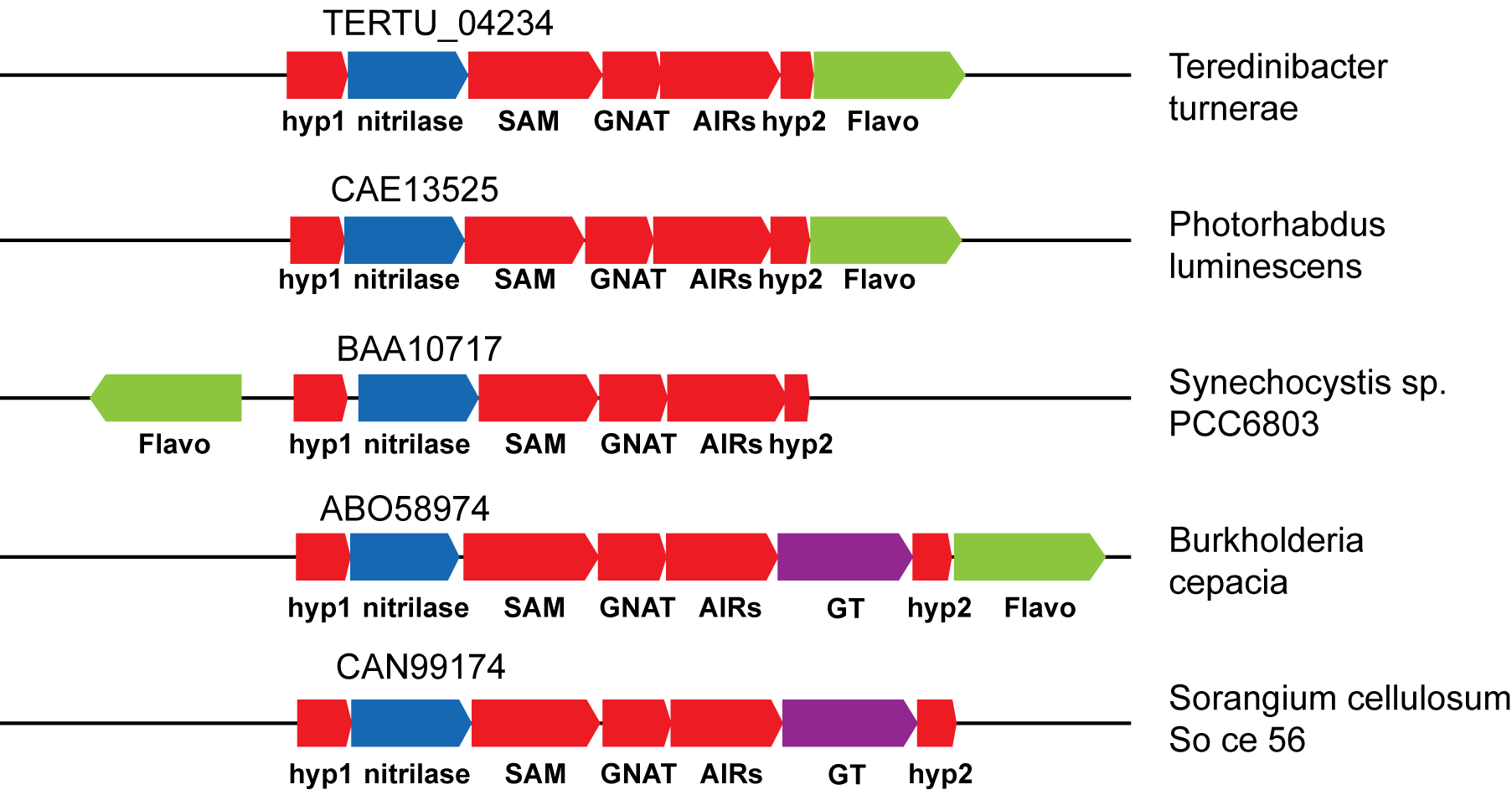

Supplement: Figure S2 — Nit1C gene cluster organization. The genomic context of the T. turnerae nitrilase gene (TERTU_04234) is shown along with similar nitrilase operons (Nit1C) from other bacterial genomes. Other proteins encoded by genes commonly found in Nit1C clusters include 2 hypothetical proteins (hyp1 and hyp2), a radical SAM superfamily protein (SAM, Pfam 04055), GCN-5 related acetyltransferse (GNAT, Pfam 00583), 5′-phosphorybosyl-5-aminoimidazole synthase-related proteins (AIRS, Pfam 00586), and a putative flavoprotein (Flavo). (0.29 MB TIF) [file pone.0006085.s007.tif]

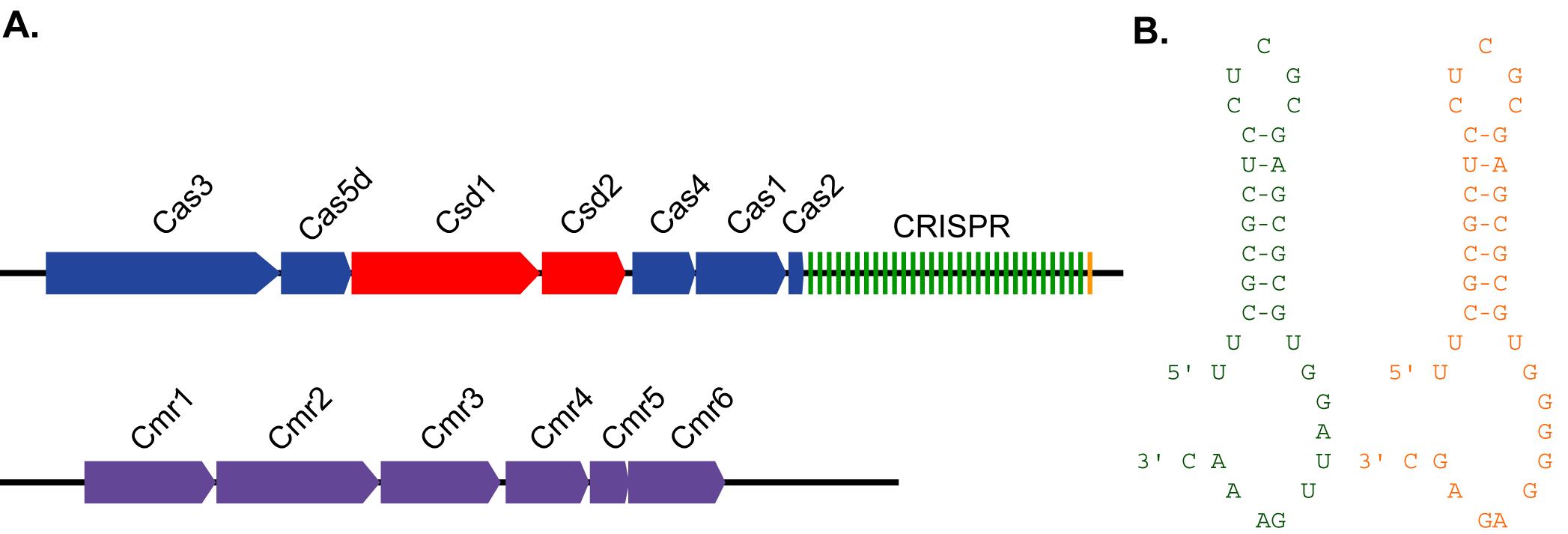

Supplement: Figure S3 — CRISPR associated genetic loci in Teredinibacter turnerae. The CRISPR associated cas/csd and cmr loci are shown (A). Genes belonging to different gene families are distinguished by color (blue, cas; red, csd; purple, cmr). The predicted CRISPR repeat RNA hairpin structure is shown (green) with the variant terminal repeat (orange). Hairpin sequences are oriented with respect to the cas operon, which is antisense to the genome. (0.19 MB TIF) [file pone.0006085.s008.tif]
